# Arctic bacterial diversity and connectivity in the coastal margin of the Last Ice Area

**DOI:** 10.1038/s43705-023-00313-w

**Published:** 2023-09-26

**Authors:** Catherine Girard, Warwick F. Vincent, Alexander I. Culley

**Affiliations:** 1 Département de biochimie, de microbiologie et de bio-informatique & Institut de biologie intégrative et des systèmes (IBIS), Université Laval, Québec, QC Canada; 2 Centre d’études nordiques (CEN), Québec, QC Canada; 3 Groupe de recherche interuniversitaire en limnologie et en écologie aquatique (GRIL), Montréal, QC Canada; 4 Département de biologie & Institut de biologie intégrative et des systèmes (IBIS), Université Laval, Québec, QC Canada; 5 Takuvik Joint International Laboratory, Université Laval, Québec, QC Canada; 6 Present Address: Département des sciences fondamentales, Université du Québec à Chicoutimi (UQAC), Chicoutimi, QC Canada; 7 Present Address: Pacific Biosciences Research Center, University of Hawaiʻi at Mānoa, Honolulu, HI USA

**Keywords:** Microbial ecology, Biodiversity, Biogeography, Microbial ecology, Climate-change ecology

## Abstract

Arctic climate change is leading to sea-ice attrition in the Last Ice Area along the northern coast of Canada and Greenland, but less attention has been given to the associated land-based ecosystems. Here we evaluated bacterial community structure in a hydrologically coupled cryo-ecosystem in the region: Thores Glacier, proglacial Thores Lake, and its outlet to the sea. Deep amplicon sequencing revealed that *Polaromonas* was ubiquitous, but differed genetically among diverse niches. Surface glacier-ice was dominated by Cyanobacteria, while the perennially ice-capped, well-mixed water column of Thores Lake had a unique assemblage of Chloroflexi, Actinobacteriota, and Planctomycetota. Species richness increased downstream, but glacier microbes were little detected in the lake, suggesting strong taxonomic sorting. Ongoing climate change and the retreat of Thores Glacier would lead to complete drainage and loss of the lake microbial ecosystem, indicating the extreme vulnerability of diverse cryohabitats and unique microbiomes in the Last Ice coastal margin.

## Introduction

The Canadian Arctic Archipelago (CAA), one of the most glaciated areas of the Arctic, has experienced rapid ice attrition over the past decades, especially at the coastal margin of the Last Ice Area (LIA). Wind and currents push the sea ice up against the coast of the far northern islands in the CAA, resulting in some of the thickest sea ice of the Arctic Ocean [1] and persistent cold conditions for habitats on and near the land such as ice shelves, glaciers and perennially ice-capped lakes. The LIA is therefore considered an enduring refuge and conservation site for polar biota, including microbes, in the face of global warming [2]. However, over the last 30 years, ice shelves in the area have contracted by 42%, glaciers have shrunk measurably, and multiyear landfast ice at this margin, some of it 50 years old, has almost completely disappeared [3]. In the coastal lands adjoining the LIA, lakes that were previously capped by thick ice have recently experienced complete ice break-up and loss in summer, causing disruption of these ecosystems by altered stratification and mixing regimes [4]. Climate-driven change in the marine environment therefore has repercussions for adjacent terrestrial cryohabitats, and for freshwater ecosystem stability.

Ice habitats in Arctic marine and terrestrial environments are known to harbor complex microbial communities [5, 6] and rapid microbial evolutionary processes [7]. Degradation of the cryosphere therefore threatens its unique biodiversity and may release ice-dwelling microbes from glaciers [8], permafrost [9] or snow [10] into downstream habitats, resulting in interactions with unknown consequences. Certain taxa such as the proteobacterial genus *Polaromonas* are known to thrive in these cold environments [11, 12], with some evidence that there may be local adaptation to exploit specific habitats [10, 13]. However, little is known about microbial diversity and connections among ice-dependent habitats in the far North and their sensitivity to change, notably in the terrestrial cryosphere and the associated meltwater habitats. High Arctic freshwaters exhibit the greatest sensitivity to rapid, warming-enduced state shifts [14], and are likely to be transformed dramatically by changes in their adjacent cryosphere.

**Fig. 1:**
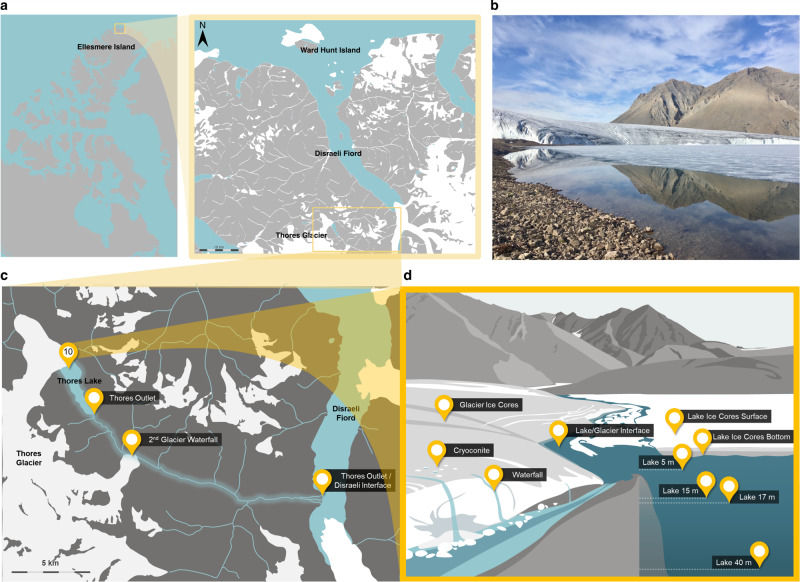
Sampling sites in the Thores glacier-lake-outflow continuum. **a** Location of the Thores system on northern Ellesmere Island, near Disraeli Fiord (inset). **b** Interface between Thores Glacier and Thores Lake. **c** Map of sampling sites collected along flow order in Thores watershed and outflow (*n* = 14). **d** Samples were collected at each site either from ice (glacier, lake ice) or water (lake, river, meltwater).

Here, we evaluated the bacterial diversity of proglacial Thores Lake (Fig. 1), an ice-dammed waterbody that lies 50 km inland from the Arctic Ocean, and 16 km upstream of a fiord in the LIA [15]. Unlike many other lakes in the region, it appears to be buffered by change as evidenced by its adjacent glacier, which is among the more stable of northern Ellesmere Island glaciers [16]. Ice-dammed lakes like Thores interact with their adjoining glaciers, and the ice-contact zone is the site of meltwater exchanges and thermal feedback [17]. Understanding the processes underway in these transition zones of proglacial lakes is critical to predicting the fate of land-terminating glaciers, and can also provide valuable information about the release of ice-dwelling organisms into the hydrosphere.

We hypothesized that cold-dwelling *Polaromonas* would be widely distributed across the Thores glacier-lake-outflow continuum, but that the diversity of cryohabitats in this system would favor niche adaptation within the genus. We further surmized that despite this differentiation, Thores Lake and its outflow would show evidence of connectivity to the cryosphere, with a strong imprint of glacier and lake ice microbes on aquatic communities. Such connectivity has been observed in a snow-lake-outflow system [10] on Ward Hunt Island, which has been impacted by climate change at the LIA coast [18]. We addressed these hypotheses by accessing the remote Thores Lake area by helicopter in mid-summer and sampling the glacier surface, the interface zone with the lake, sites throughout the lake, perennial lake ice, and the river outflow at its outlet and 16 km downstream, just before its entry into the fiord. Deep amplicon sequencing of these samples allowed us to identify similarities and differences in bacterial community structure, and the niche separation of *Polaromonas* phylotypes across this coupled ice-water ecosystem in the LIA terrestrial margin.

## Material and methods

### Site description

Thores Lake (82.65162°N, -73.663669°W) is an ultraoligotrophic, unstratified proglacial lake [15] located in remote Quttinirpaaq National Park on Ellesmere Island, Nunavut, Canada. The lake has a maximum depth of at least 69 m and an area of 2.4 km^2^. It is fed by Thores Glacier, which is stable in contrast to many other glaciers on Northern Ellesmere Island [16], and is drained by Thores River into Disraeli Fiord and the Arctic Ocean [15, 16].

### Sample collection

Samples were collected in summer (July-August) 2018 and 2019, at the sites shown in Fig. 1. Physico-chemical parameters were measured using an RBR*concerto*^3^ profiling system (RBR Ltd, Ottawa, Canada) that measured temperature, conductivity, chlorophyll-a, dissolved oxygen, and depth. All samples in the study were collected in triplicate. Detailed sampling methods and water chemistry data are presented in Culley et al. [15].

Three 30 cm diameter holes were bored 10 m apart through the ice cover on Thores Lake (115 cm ice thickness). Lake water samples were collected using a 7-liter Limnos sampler at depths of 5, 15, 17, and 40 m, and stored in low-density polyethylene Cubitainers that had been washed with 2% (vol/vol) Contrad 70 liquid detergent (DeconLabs, King of Prussia, PA, USA) and 10% (vol/vol) ACS-grade HCl (Sigma-Aldrich, St. Louis, MO, USA) and rinsed with lake water. Flowing water (rivers, streams, waterfalls) was collected as grab samples into Contrad and HCL-washed Nalgene high density polyethylene (HDPE) bottles. Ice samples were collected with a Mark II corer (Kovacs Enterprise, Roseburg, OR, USA) from lake ice and the glacier, and thawed in sterile 5-liter Whirl-Pak^TM^ bags (Whirl-Pak, Madison, WI, USA) in the dark at 4 °C. Snow samples were also collected into opaque HDPE bottles, and thawed in the dark at 4 °C.

Subsamples from water and thawed ice samples were stored at 4 °C in the dark for analysis of total phosphorus (TP), total nitrogen (TN), and dissolved organic carbon (DOC). Subsamples were also filtered onto 25-mm diameter Whatman® GF/F glass-fiber filters (nominal pore size of 0.7 µm) (Sigma-Aldrich) and immediately frozen at −20 °C for pigment analyses. Whole water for sequencing was filtered separately onto Sterivex™ 0.22 µm capsule filters (Millipore, Burlington, MA, USA) in 2018 and onto Anotop® 0.02 µm syringe filters (Whatman, Maidstone, United Kingdom) in 2019. Filters were stored at −20 °C on the field, and at −80 °C at Université Laval until nucleic acid extraction.

Sampling dates and filters used for molecular work are presented in Supplementary Table S1.

### Sample processing

TP, TN and DOC samples were analyzed at l’*Université du Québec à Montréal* (UQAM, Montréal) [19–21]. Fatty acids were analyzed by the *Fatty Acid Analytical Laboratory* (UQAC, Chicoutimi) as described in Wauthy & Rautio [22]. Pigments were analyzed by high-performance liquid chromatography (HPLC), as in Thaler et al. [23]. Water chemistry results for are reported in Supplementary Table S2, and full water chemistry data set and RBR profiles are available online in the Nordicana environmental data archive [24, 25].

Nucleic acids were extracted from Sterivex™ filters with a modified version of the AllPrep DNA/RNA Mini kit (Qiagen, Hilden, Germany), as described in Cruaud et al. [26] and 0.02 µm Anotop® filters were extracted with the MasterPure Complete DNA & RNA purification kit (Epicentre, Madison, WI, USA) using the backflushing technique described by Mueller et al. [27]. Libraries were prepared by 2-step PCR targeting the V4 region of the 16 S rRNA gene (with primers 515 F & 806 R) with the Q5 High-fidelity polymerase (NEB, Ipswich, MA, USA), purified on SparQ Purmag beads (QuantaBio, Beverly, MA, USA) and sequenced on an Illumina MiSeq by paired-end sequencing (2x300bp) at the *Institut de biologie intégrative et des systèmes* (IBIS, Université Laval). Sequencing yielded a total of 8 845 831 reads (see Supplementary Table S3 for DNA concentration and sequencing yield per sample).

### Sequence processing and statistical analyses

Of the 19 samples sequenced, 5 were removed from the subsequent analyses due to low read counts (<14 000 reads, all others between 255 000-380 000 reads; Supplementary Table S3). Reads were processed with DADA2 (v1.14.0) [28] in R (v3.6.1) [29] for quality-filtering, paired-end concatenation, removal of chimeras and identification of exact amplicon sequence variants (ASVs) [30]. This was done using default parameters except in the *filterAndTrim()* function for *truncLen* (275,250) and *trimLeft* (19,20), chosen from the error rate quality graphs. Taxonomy was assigned using SILVA (release 138) [31] and chloroplast-assigned sequences were removed, leaving a total of 6 785 different ASVs across 14 samples. Taxonomy was assigned at least to phylum level for 95.9% of reads. These 14 samples (each containing between 111 707 and 200 611 quality-controlled reads per sample) are presented throughout the manuscript in the order of flow, from upstream habitats to downstream sites (Fig. 1c). For read processing throughout the DADA2 pipeline, see Supplementary Table S3.

ASVs were analyzed in R with the phyloseq{} package (v1.30.0) [32]. A raw ASV table was used for community composition figures, calculating core communities, biomarker analyses, source tracking, and for alpha diversity estimates, while a table transformed by Hellinger standardization with the *decostand()* function in vegan{} (v2.5-6) [33] was used for beta diversity and ordinations. ASV table manipulations were done using the stringr{} (v1.4.0) [34], DECIPHER{} (v2.14.0) [35], tibble{} (v3.0.2), dplyr{} (v1.0.0) [36] and tidyr{} (v1.1.0) [37] packages in R.

Alpha diversity estimates were calculated with Chao1 and Shannon’s diversity indices with phyloseq{}. Community composition was plotted with ggplot2{} [38] and RColorBrewer{} (v1.1-2) [39], and core microbiome members were identified with the *core()* function in the microbiome{} package (v1.8.0) [40] (taxa detected in >50% of samples at a relative abundance >1%). Community diversity was calculated using Bray-Curtis’ dissimilarity index and plotted as ordinations in Principal Coordinate Analysis (PCoA) space with phyloseq{}. Analysis of variance comparing habitat type (ice, lake, river) was calculated with the *adonis()* function, and group dispersion homogeneity was verified with *betadisper()* in vegan{} (*P* = 0.153, *F* = 2.2537, permutations = 999). Photosynthetic oxygen regimes (oxygenic, anoxygenic) were assigned to taxa based on known photosynthesis metabolism in bacteria [41]. Anoxygenic organisms grouped all photosynthetic organisms that did not belong to the Cyanobacteria phylum, and anoxygenic and oxygenic organisms were plotted by relative abundance.

Discriminating features (biomarkers) in ice and water samples were identified with linear discriminant analysis (LDA) effect size (LEfSe) [42] implemented in microbiomeMarker{} (v.0.0.1.9000) [43] (LDA cutoff = 4, Kruskal–Wallis *P* value cutoff = 0.05, bootstrap = 30). Intersections between *Polaromonas* in Thores glacier-lake continuum were plotted with UpSetR{} (1.4.0) [44]. The source of ASVs along the flow order was identified by Bayesian approach using SourceTracker [45] (sources = glacier, lake ice, lake water; sinks = lake, lake ice, outflow) (α = 0.001, with mean prediction proportion and standard deviation reported from Gibbs sampling over 10 draws).

The taxonomy of *Polaromonas*-assigned ASVs was inferred through phylogeny, using partial 16 S sequences from isolates of the representatives of the nine *Polaromonas* species (*P. naphtalenivorans* CJ2 [NR_027567.1], *P. hydrogenivorans* strain DSM 17735 [NR_043540.1], *P. glacialis* strain Cr4-12 [NR_109013.1], *P. cryoconiti* strain Cr4-35 [NR_109012.1], *P. jejuensis* strain JS12-13 [EU030285.1], *P. aquatica* strain CCUG 29402 [NR_042404.1], *P. ginsengisoli* strain Gsoil 115 [AB245355.1], *P. vacuolata* strain 34-P [NR_025958.1], *P. eurypsychrophila* strain B717-2 [NR_149767.1]). The phylogenetic tree of these species was computed with ClustalOmega [46], rooted with a *Rhodoferax* (closely related genus [47]) ASV from this study. The tree was plotted and annotated using iTol (v4) [48].

*Polaromonas* sequences from cryospheric habitats were collected in fasta format from the NCBI Nucleotide database using esearch() and efetch() from the E-utilities package (NCBI) by using the query “txid52972[Organism]” in combination with either of the following keywords: “ice”, “glacier”, “Arctic” and “Antarctica”. This produced a set of 855 unique *Polaromonas* sequences. From these sequences, whole genomes as well as plasmids were removed, and we retained only sequences from the 16 S rRNA gene (376 sequences). To these we added the 16 S sequences from uncultured bacterium reported to be *Polaromonas* identified by Darcy et al. [11] (55 sequences) as well as the 12 ASVs from Northern Ellesmere identified as *Polaromonas* in our pipeline. These sequences were aligned with mafft (v7.453) [49], the alignment was trimmed to the consensus region of 286 bp with MegaX (v10.1.8) [50], and non-aligning sequences were removed with SeaView (v5.0.4) [51], producing a final data set of 363 *Polaromonas* 16 S genes or gene fragments. The phylogenetic tree was produced as described in the previous paragraph. For accession numbers and references of sequences obtained on NCBI, see Supplementary Table S4.

## Results

### *Polaromonas* ubiquity and niche separation

**Fig. 2:**
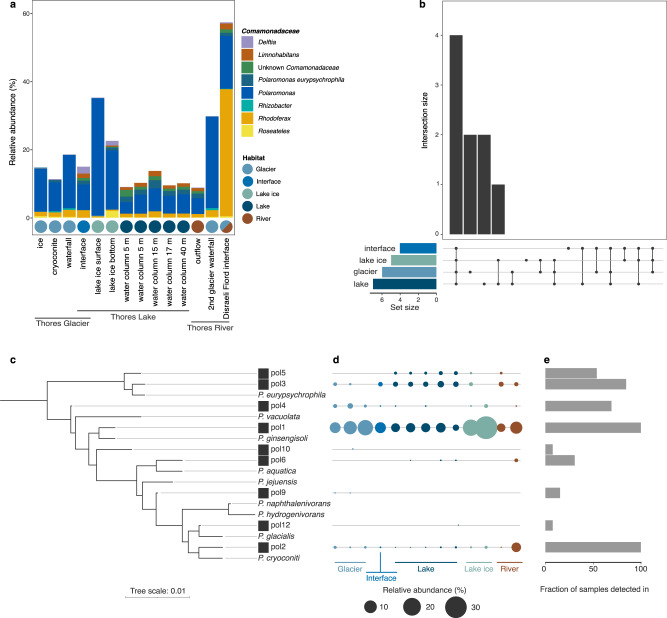
Distribution and phylogenetic diversity of *Polaromonas* phylotypes. **a** Relative abundance of main genera of the *Comamonadaceae* family, including *Polaromonas*. **b** Proportion of shared *Polaromonas* in the glacier-lake continuum between interface, lake ice, glacier, and lake water habitats (with the number of ASVs in each habitat in parentheses). Most *Polaromonas* phylotypes were shared across all four compartments, while the glacier and lake had 2 unique phylotypes each. Lake and lake ice shared one phylotype. **c** Phylogenetic tree of Thores *Polaromonas* ASVs and partial 16 S sequences of previously isolated representatives of the nine *Polaromonas* species (*P. eurypsychrophila* NR 149767.1, *P. vacuolata* NR 025958.1, *P. ginsengisoli* AB245355.1, *P. aquatica* NR 042404.1, *P. jejuensis* EU0302285.1, *P hydrogenivorans* NR 043540.1, *P. cryoconiti* NR 109012.1, *P. glacialis* NR 109013.1, *P. naphthalenivorans* NR 027567.1). The tree is rooted with a *Rhodoferax* ASV from the Thores dataset. **d** The relative abundance of each Thores *Polaromonas* phylotype is shown by the size of the circle, which is organized by sample type (order and color) is represented by sample type. **e** Fraction of samples in which each Thores *Polaromonas* was detected. The waterfall flowing from another glacier downstream of the lake was omitted from this analysis (a total of 13 samples).

The core microbiome across the Thores glacier-lake-outflow system contained 141 amplicon sequence variants (ASVs), with around one tenth belonging to the *Comamonadaceae* (4 *Polaromonas* ASVs, 4 *Rhodoferax*, 1 *Limnohabitans*, 1 *Delftia*, 1 *Roseateles* and 3 unidentified *Comamonadaceae*; Supplementary Table S5). This family represented a substantial proportion of all reads from the Thores watershed (between 9 and 59% of reads per sample, average ± SD of 19 ± 14%). *Polaromonas* were significantly more characteristic of ice than water samples (LDA score = 5.10, *P* = 0.004, Supplementary Table S6 & Fig. 2a). This genus was detected throughout the glacier-lake-outlet ecosystem (Fig. 2a), outnumbering all other members of the *Comamonadaceae* (except *Rhodoferax* at the junction between the outlet and Arctic Ocean in Disraeli Fiord), and represented 19% of reads in the cryosphere samples and 8.2% of lake sequences.

While most *Polaromonas* ASVs were common to all compartments of the glacier-lake continuum (Fig. 2b), there was inter-habitat diversity within the genus (Fig. 2c, d). One *Polaromonas* ASV (pol1) dominated other members of the genus, accounting for over 25% of reads per sample (Fig. 2c, d). This phylotype, which was closely related to *P. ginsengisoli* (Fig. 2c) was ubiquitous throughout the continuum (Fig. 2e). Another *Polaromonas* phylotype (pol2), closely related to *P. cryoconiti*, was also detected in all compartments, but at much lower relative abundances (5.5% of ASVs in the junction with Disraeli Fiord, maximum of 0.5% of reads in all other samples) (Fig. 2e). Two phylotypes (pol3 & pol5) related to *P. eurypsychrophila* were most abundant in the lake’s water column, although detected in lower abundances in other compartments, similarly to pol6, related to *P. aquatica* (Fig. 2d). Two low abundance phylotypes (pol9 & pol10), related to *P. aquatica* and *P. naphthalenivorens* (Fig. 2d), were unique to the glacier (Fig. 2b). All phylotypes identified in lake ice were shared with the water column (Fig. 2b), and the interface had no unique *Polaromonas* phylotypes – all representatives identified in this compartment were shared with the glacier, water column and lake ice (Fig. 2b).

**Fig. 3:**
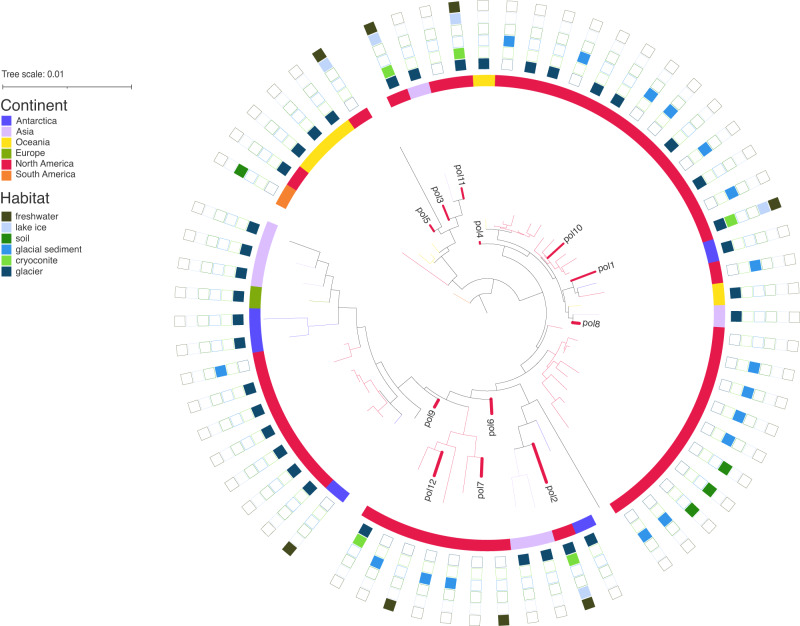
Global phylogeny of *Polaromonas* sequences. Phylogeny of *Polaromonas*-identified ASVs from the Thores system, aligned along the V4 region of the 16 S rRNA gene of *Polaromonas* isolates from Ward Hunt Island (NEI) and around the globe accessed through NCBI. For accession numbers and metadata for isolates, see Supplementary Table S4.

A meta-analysis including sequences from six continents and multiple habitat types showed that the most prevalent *Polaromonas* phylotype in our analyses (pol1), i.e. the ASV present in the greatest fraction of samples and at the greatest abundance (Fig. 2d, e), was most closely related to isolates from Collins Glacier (Antarctica) and Arikaree Glacier (Colorado) (Fig. 3). Thores *Polaromonas* phylotypes did not group together, and lake phylotypes (pol3 & pol5) grouped closer to the root of the tree (Fig. 3). The pol12 phylotype (which was related to *P. glacialis* in the reference tree (Fig. 2c)) grouped with North American glaciers and lakes.

### Cryohabitat diversity and bacterial assemblages

**Fig. 4:**
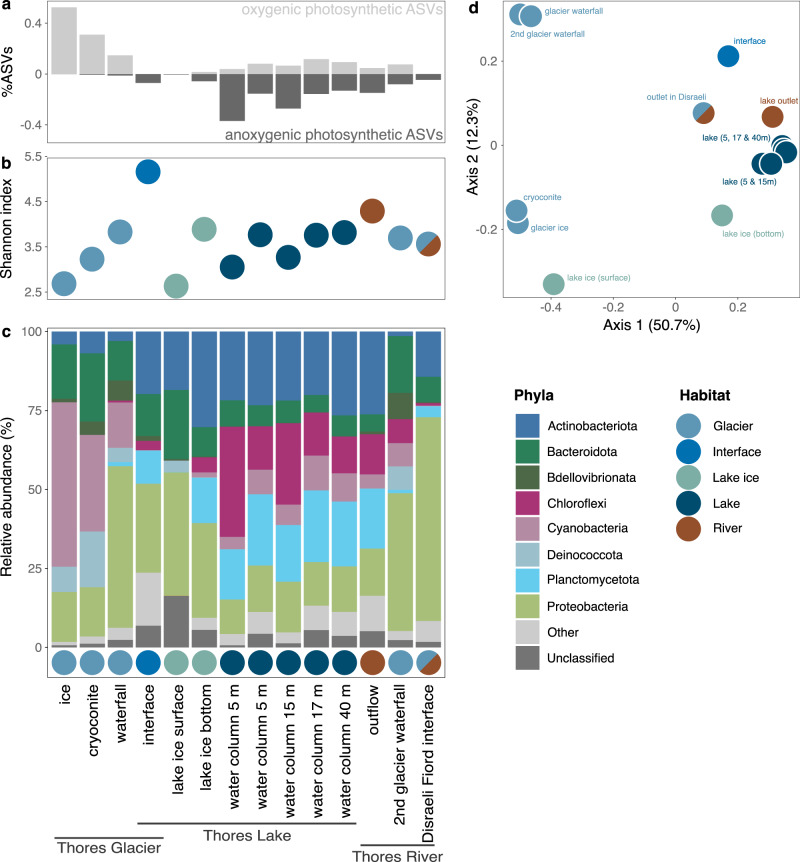
Microbial diversity across the Thores glacier-lake-outflow continuum. **a** Relative abundance of photosynthetic organisms based on their oxygen regime. **b** Sample richness (from Shannon’s diversity index). **c** Phylum-level relative abundance (top dataset-wide phyla shown, the rest grouped in “Others”). Colors show habitat type – when a sample was taken at the interface of two categories, both colors are represented. **d** Principal coordinate analyses from Bray-Curtis dissimilarity matrix showing beta diversity across all samples.

The Thores lake-glacier continuum encompassed different types of cryospheric habitats, including glacier ice, glacier-associated habitats such as cryoconite holes, and lake ice. While *Polaromonas* dominated, they were only part of the rich assemblages identified in these cryohabitats. The Deinococcota phylum (including *Deinococcus*) (LDA score = 4.87, *P* = 0.002), Bacteroidota (including Cytophagales) (LDA score = 5.00, *P* = 0.004), Pseudanabaenales (LDA score = 4.69, *P* = 0.04) and the Bdellovibrionata order 0319-6G20 (LDA score = 4.27, *P* = 0.01) were significantly more characteristic of the cryosphere than lake and river water (Supplementary Table S6), and Deinococcota were overrepresented in Thores Glacier ice and cryoconite water. In these same samples, up to 50% of reads belonged to Cyanobacteria (Fig. 4c, Supplementary Fig. S1 & S2) and were partitioned by ice type: the filamentous, biofilm-associated taxa *Leptolyngbya* and *Pseudanabaena* dominated the glacier samples (Supplementary Fig. S1), while the bottom of lake ice contained only planktonic *Cyanobium*, suggesting its origin from lake water freeze-up. Surface lake ice (in contact with air) on the other hand contained a large proportion of sequences whose taxonomy at the phylum level could not be assigned (16.3%, Fig. 4c). Waterfalls flowing from Thores Glacier, and from a second, unnamed glacier 6.3 km downstream into Thores River were similar to each other in composition (Fig. 4d), but distinct from the glacier ice and cryoconites: both waterfalls had more *Comamonadaceae* (Proteobacteria) and *Pseudanabaenaceae* (Cyanobacteria) than ice (Supplementary Fig. S2).

### Distinct community composition in downstream aquatic habitats

**Fig. 5:**
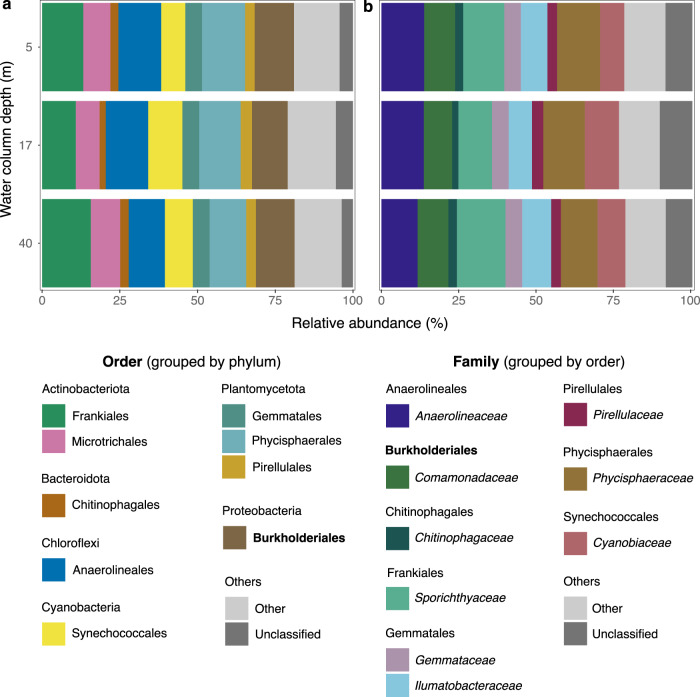
Taxonomic composition of the Thores Lake water column. **a** Order composition of taxa accounting for >2% of reads from 0.02 µm Anotop filters, with Burkholderiales (to which *Polaromonas* belong) in bold. **b** Family composition of taxa accounting for >2% of reads, with *Comamonadaceae* (to which *Polaromonas*) in bold.

The planktonic communities in Thores Lake differed strikingly from the glacial communities (Fig. 4c, d). The reads were dominated by *Ilumatobacteraceae* and *Sporychtyaceae* (Actinobacteriota), *Anaerolineaeceae* (Chloroflexi), *Gemmataceae*, *Phycisphaeraceae* and *Pirellulaceae* (Planctomycetota) (Fig. 5, Supplementary Fig. S2) and were homogenous to 40 m, consistent with the lack of thermal or oxic stratification in Thores Lake in the sampling depth range. *Comamonadaceae* (including *Polaromonas*) were also consistently present throughout the water column (average of 9.1% of reads; Fig. 5). Bacterial phototrophs in the water column were mostly associated with anoxygenic photosynthesis (Choroflexi), contrary to glacier prokaryotes which were overwhelmingly linked to oxygenic photosynthesis (Cyanobacteria) (Fig. 4a). However, oxygenic Synechococcales were detected in low relative abundance in the water column, and were the only representatives of Cyanobacteria in the lake (Fig. 5 & Supplementary Fig. S1).

The ice-contact zone with its unstable ice cover and high turbidity connecting the glacier and lake (hereafter referred to as the *interface*) was dominated by Proteobacteria (Fig. 4c), and was the most diverse site across the Thores glacier-lake-outflow continuum (Fig. 4b, 981 taxa), with a mixture of glacial and lake water representatives. Near its outflow from the lake, the Thores River community was similar to the lake plankton assemblage, and this community composition persisted 16 km downstream at its entry point to Disraeli Fiord, but with an increased representation of *Comamonadaceae* (Proteobacteria), and a decreasing proportion of unassigned taxa (Supplementary Fig. S2).

### Bacterial connectivity between habitats

Habitat type explained 46.3% of variation in community composition (adonis *P* = 0.001, *F* = 4.739, degrees of freedom = 2), with cryosphere-associated samples clustering along Axis 1, except for the bottom of lake ice (Fig. 4d). The interface was distinct from the glacier and water column, and with some resemblance to lake ice (Fig. 4d), had the greatest taxonomic richness (Fig. 4b) and no unique *Polaromonas* phylotypes, unlike the glacier and lake (Fig. 2b). While samples were collected over two summers using different filter sizes (0.22 and 0.02 µm) and corresponding nucleic acid extraction protocols (see Methods), filter type contributed only marginally to explaining community assemblage (21% variation, *P* = 0.01) (Supplementary Fig. S3).

**Fig. 6:**
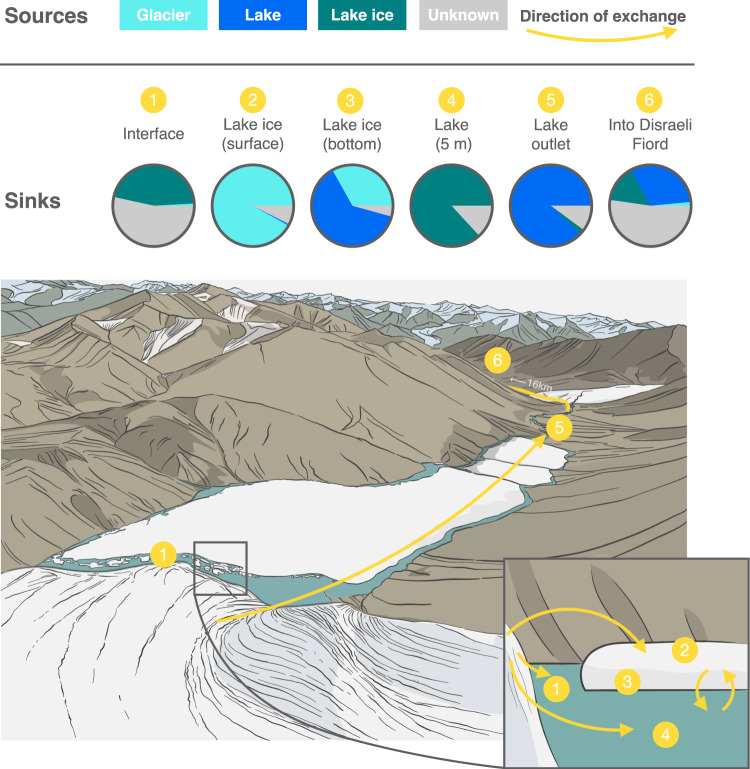
Source tracking of microbial taxa in the Thores glacier-lake-outflow continuum. Potential sources are the glacier, lake ice, and lake water. Taxa whose origin could not be traced to these categories are marked as “Unknown”. Sinks across the continuum include the interface, lake ice (surface and bottom), lake water (at 5 m), the mouth of the outflow, and its junction in Disraeli Fiord 16 km downstream. Pie charts display the proportion of the origin of microbial taxa in each sink, and yellow arrows show the direction of exchanges from sources to sinks across the continuum. Circled numbers identify sinks on the illustrations. The mean source tracking values and confidence intervals for all sinks (including the rest of the water column) are given in Supplementary Table S7.

Source tracking identified 47% of taxa in the glacier-lake interface as originating from lake ice (Fig. 6, Supplementary Table S7), but the origin of most taxa in this sample could not be determined. However, the proportion of identifiable taxa rose to 90% in Thores River. The lake water column and ice cover appeared to influence one another: 87–93% of water column microbes were tracked to ice, and 62.6% of microbes in the bottom layers of lake ice were sourced to the water column below (Fig. 6 & Supplementary Table S7). Surface lake ice was more impacted by the glacier (91.9%), and little seeded by the water column at the time of sampling (0.6%) (Fig. 6 & Supplementary Table S7). At the head of Thores River, approximately 88% of taxa were from the lake, and at its mouth near Disraeli Fiord, 16 km downstream, 32% of taxa could still be tracked to Thores Lake.

## Discussion

Our observations from the LIA terrestrial margin draw attention to the rich diversity of cryohabitats and bacterial communities within this region and their vulnerability to climate change. The genus *Polaromonas* was found across almost all habitat types, and consistent with our hypothesis of genetic divergence among niches, there were pronounced differences in *Polaromonas* phylotypes between the ice and water environments. This niche specificity extended to the overall communities, but contrary to our expectation of hydrological connectivity as a dominant force in downstream assembly processes, the bacterial dominants diverged greatly between the glacier and the main body of the lake. The ice margin interface had its own distinctive microbiome, with a mixture of the lake and glacial *Polaromonas* phylotypes, but also with the highest diversity of other bacterial taxa, including many that were not shared upstream with the glacier nor downstream in proglacial Thores Lake and its outflow.

Cold-dwelling *Comamonadaceae* are widely distributed in the Arctic, including in thermokarst thaw ponds [52] and in Lake Hazen [53], a large lake on Ellesmere Island that lies 100 km southeast of Thores Lake. *Polaromonas* was the most notable member of the *Comamonadaceae* family in this study, and one *Polaromonas* phylotype was the only taxon present in all samples in the dataset (at a 1% relative abundance threshold). This genus is ubiquitous in the polar regions and other cold environments [13] and has an especially high potential for dispersal [11], consistent with the presence of shared *Polaromonas* ASVs between habitats in the Thores glacier-lake-river continuum.

Many of the *Polaromonas* phylotypes identified in Thores were generalists, i.e. organisms that appear to survive under diverse conditions, consistent with the metabolic versatility found within this genus [54]. This includes phylotype pol2, which was detected throughout the watershed, with the greatest relative abundances at the junction of Thores River in Disraeli Fiord. Phylotype pol1, which was the most abundant member of the *Polaromonas* genus in Thores, was also ubiquitous. This phylotype was closely related to *P. ginsengisoli*, which was initially isolated from soil, and shares 98.6% of its 16 S rRNA with *P. eurypsychrophila* [55], but has since been identified in other systems, including in the water column of a boreal lake during winter [56]. The consistent presence of these two phylotypes (pol1, pol2) across the watershed suggests these members of the *Polaromonas* genus are generalists with broad tolerances that likely endure year-to-year, as data presented here were collected over two consecutive summers.

Phylotypes pol3 & pol5 were consistently found in the lake, as well as in lesser abundances in other compartments downstream of the lake. These organisms were closely related to *P. eurypsychrophila*, which was originally isolated in glacier ice on the Tibetan Plateau [57]. These phylotypes were either not detected, or present in low abundances in Thores glacier ice. Different drivers of niche-partitioning of *Polaromonas* have been suggested to operate on glacial surfaces, including sorting by local habitat conditions, but also community interactions with other prokaryotes and with eukaryotic microbes [13], which may also be at play in free-flowing waters in the Thores system.

The lack of *Polaromonas* phylotypes specific to the glacier-lake interface implies that this is a mixing and transition zone, with insufficient time or selective forces to result in detectable genetic differentiation within this taxon. A limnological study of Thores Lake showed that this zone has many distinct properties, including in nutrients, conductivity and turbidity [14]. Consistent with these features and with the many bacterial ASVs (but not *Polaromonas*) that were observed in the present study to be unique to this zone, the interface also contained a distinct phytoplankton community, with a higher proportion of green algae and the chlorophyte pigment chlorophyll *b* relative to the main body of the lake, which was characterized by chrysophytes and fucoxanthin [15]. We found that the interface community lacked the filamentous cyanobacteria *Leptolyngbya* and *Pseudanabaena* that dominated the glacier samples, indicating strong selection against them, or reduced dispersal abilities of these biofilm-forming taxa. As sampling occurred over two different summers, there may be inter- or intra-year variation patterns that were not observed. However, Cyanobacteria have been found to exhibit long-term persistence in perennially cold habitats, suggesting robust assemblages on the glacier surface [58].

The presence of both lake and glacier *Polaromonas* in the mixing zone suggests that these phylotypes have sufficiently broad tolerances to persist in such conditions, and to resist microbial loss processes during their short residence time in the transition zone, while other groups like Cyanobacteria that originate from the glacier are more likely to be filtered in the interface.

Source tracking has been used in many instances to model microbial connectivity across glaciers [8, 59, 60], and has been applied in nearby Ward Hunt Lake [10], where snow was found to be a major seeder of microbes to the lake, including *Polaromonas* in the littoral mixing zone. In the present study, we were unable to identify the source of most taxa, especially in the lake, and the percentage of taxa whose source was identified was lower than at Ward Hunt. This may be due to the long term stability of Thores Glacier [16] and its specialized microbiome, or to the longer residence time of Thores Lake [15] that allows sufficient time for in-lake processes to shape the communities.

The strong in-lake effect on microbiome composition was indicated not only by the unique *Polaromonas* phylotypes in Thores Lake but also by the distinctive assemblage of other taxa. Actinobacteriota accounted for approximately 20% of reads throughout the water column; cells of this phylum are typically small in size and are often abundant in oligotrophic freshwaters [61]. Planctomycetota (average of 19.8% of lake reads) has also been identified as an important feature of ice-covered lakes at the far northern coast of Ellesmere Island [62], and one of the main planctomycete families in Thores Lake, the *Gemmataceae*, is known to include psychrotolerant taxa [63]. The high relative abundance (13%) of Anaerolineales (Phylum Chloroflexi) is also unusual for most freshwater ecosystems and implies the importance of anoxygenic aerobic phototrophy in Thores Lake. This class has been identified among the dominants in deep, cold, oxygenated hypolimnia, including in Lake Biwa [64], Japan, and Crater Lake [65], USA. These distinctive microbiome features of Thores Lake also differed markedly from High Arctic Lake Hazen, where ice has a less pervasive influence on habitat structure. *Polaromonas* was only detected at the bottom of Lake Hazen where glacial meltwater joins the water column [53], whereas this genus was ubiquitous throughout Thores Lake. Furthermore, while Chloroflexi were an important feature of Lake Hazen in spring at comparable relative abundances to Thores Lake, they decreased over summer [53]; and Bacteroidota, the second most abundant phylum in Lake Hazen, accounted for less than 6% of reads in the Thores water column.

Another cryospheric compartment of interest in the Thores Lake system is the perennial ice cover, which would protect the lake from direct airborne seeding. There are likely, however, to be complex interactions between the lake ice and the underlying lake waters. While dissolved compounds (organic carbon, phosphorus) are excluded from lake ice during its formation, particles can be retained in the new ice [66]. This freeze-trapping effect may explain the presence of *Cyanobium* in the bottom of Thores Lake ice since this picocyanobacterial genus was abundant in the surface waters of the lake.

The results obtained here underscore the high bacterial species richness in the Thores ice-water ecosystem, and the diverse array of community assemblages that segregate among cryohabitats. The presence of ASVs does not necessarily imply that the cells are actively growing, but transcriptomic studies of glacier microbiomes elsewhere have indicated that a large proportion of the observed community has the potential for metabolic activity [67]. However, our analyses captured only a subset of the total microbial diversity, and studies at other ice-associated sites in the LIA margin have drawn attention to diverse assemblages of archaea [68], viruses [69, 70], and microbial eukaryotes, including new fungal taxa [71]. Microbial surveys of cryohabitats in other regions have shown the importance of desmid blooms on ice caps [72] and snow algal growth on glaciers [73]. These components are likely to be similarly diverse in the Thores ecosystem, and to be similarly vulnerable to any habitat loss that accompanies ongoing climate warming in the region. While it is challenging to obtain high-resolution data in systems as remote as Thores, this study provides a snapshot of the microbial assemblages of a rare, stable glacier-lake coupled system in the High Arctic. There is a pressing need for further research to increase the spatial and temporal coverage of microbiome studies in polar environments.

Arctic ice-dwelling microbes are at the forefront of climate change, and are threatened by habitat extinction. There is therefore an urgent need to understand this unique biodiversity before it is lost to further warming [74, 75]. Microbes may also accelerate the melting of ice in warmer conditions, by reducing albedo and promoting ablation of glaciers, snow, and ice shelves [73, 76]. Shrinking of the cryosphere will increase the dispersal of microbes across Arctic landscapes [77–79], and while some microbes will be driven to extinction by the environmental conditions in their new habitats, cold-adapted *Polaromonas* may evolve into novel ecotypes, potentially losing cold-adaptation functions and genetic diversity.

The Last Ice Area is projected to remain ice-covered into the middle of the century when summer ice will have largely disappeared from the rest of the Arctic Ocean [1]. The perennially cold terrestrial margin will therefore be an enduring refuge for ice-dependent microbes in the coastal High Arctic. However, proglacial ecosystems such as the Thores glacier-lake-outflow continuum are entirely dependent on the integrity of the glacial ice, and loss of the glacier would result in complete drainage and loss of the lake and its associated environments [16]. Cryohabitats and their rich microbiota, including specialized ecotypes of *Polaromonas*, will likely persist in this refuge into the immediate future, but will be prone to more severe effects of ongoing climate change.

### Supplementary information

Supplementary Information

## Data Availability

Raw amplicon sequences are available at the NCBI public database under BioProject ID PRJNA905405.
