# Supplementary material for: Arctic bacterial diversity and connectivity in the coastal margin of the Last Ice Area
Source: ISME Commun. 2023 Sep 26;3:105. doi: 10.1038/s43705-023-00313-w (PMC10522646; doi:10.1038/s43705-023-00313-w)
Supplement: Supplementary file 1 — Supplementary Information [file 43705_2023_313_MOESM1_ESM.pdf]

Arctic bacterial diversity and connectivity in the coastal margin of the Last Ice Area

Catherine Girard[1,2,3*†], Warwick F. Vincent[2,4,5], Alexander I. Culley[1,2,5 ††]
[1]Département de biochimie, de microbiologie et de bio-informatique & Institut de biologie intégrative et des systèmes (IBIS), Université Laval, Québec, Québec, Canada.
[2]Centre d'études nordiques (CEN), Québec, Québec, Canada.
[3]Groupe de recherche interuniversitaire en limnologie et en écologie aquatique (GRIL), Montréal, Québec, Canada.
[4]Département de biologie & Institut de biologie intégrative et des systèmes (IBIS), Université Laval, Québec, Québec, Canada.
[5]Takuvik Joint International Laboratory, Université Laval, Québec, Québec, Canada.

† Current affiliation: Département des sciences fondamentales, Université du Québec à Chicoutimi (UQAC), Chicoutimi, Québec, Canada
†† Current affiliation: Pacific Biosciences Research Center, University of Hawaiʻi at Mānoa, Honolulu, Hawaiʻi, USA
*** Corresponding author:** catherine5_girard@uqac.ca, https://orcid.org/0000-0002-3899-0180

Supplementary materials include the following:

Supplementary Tables

- Supplementary Table S1: Sample data
- Supplementary Table S2: Water chemistry results across all sites
- Supplementary Table S3: ASV tracking through analytical pipeline
- Supplementary Table S4: *Polaromonas* accession numbers and references (see Excel file)
- Supplementary Table S5: Core microbiome composition Thores valley (see Excel file)
- Supplementary Table S6: Habitat features (see Excel file)
- Supplementary Table S7: Source tracking of bacterial taxa in the glacier-lake-outflow

Supplementary Figures

- Supplementary Fig. S1: Cyanobacteria distribution and phylogeny
- Supplementary Fig. S2: Family-level composition
- Supplementary Fig. S3: Filter type

SUPPLEMENTARY TABLES

Supplementary Table S1. Sample data.

Sample ID	Sample description	Filter type	Sampling year
CGST40B	Thores Lake 40m	0.22µm (Sterivex)	2018
CGST5A	Thores Lake 5m	0.22µm (Sterivex)	2018
CGSTI7B	Thores Lake 17m	0.22µm (Sterivex)	2018
CGSTIB	Thores Glacier-Lake interface	0.22µm (Sterivex)	2018
CGSTOC	Thores River (lake outlet)	0.22µm (Sterivex)	2018
CG2GA	2nd Glacier melt water	0.02µm (Anotop)	2019
CG2RJA	Two-river Junction	0.02µm (Anotop)	2019
CGCA	Thores Glacier cryoconites	0.02µm (Anotop)	2019
CGT15B	Thores Lake 15m	0.02µm (Anotop)	2019
CGT2JA	Thores River and 2nd Glacier junction	0.02µm (Anotop)	2019
CGT5A	Thores Lake 5m	0.02µm (Anotop)	2019
CGTDIA	Thores River & Disraeli fiord interface	0.02µm (Anotop)	2019
CGTGCB	Thores Glacier core	0.02µm (Anotop)	2019
CGTIBA	Thores Lake ice (bottom)	0.02µm (Anotop)	2019
CGTIC	Thores Glacier-Lake interface	0.02µm (Anotop)	2019
CGTOA	Thores River (lake outlet)	0.02µm (Anotop)	2019
CGTSA	Thores Lake ice (surface)	0.02µm (Anotop)	2019
CGTWFB	Thores Glacier waterfall	0.02µm (Anotop)	2019
CGTWTB	Thores water tracks	0.02µm (Anotop)	2019

Supplementary Table S2. Nutrient, pigment and fatty acid results in water and ice samples from the Thores glacier-lake continuum. Interface in Thores refers to the point of contact between Thores glacier and Thores lake. Nutrients include total phosphorus (TP), total nitrogen (TN), nitrogen dioxide and nitrate (NO_x) and dissolved organic carbon (DOC). Pigments (chlorophylls, carotenes and xanthophylls) and fatty acids (of algal, bacterial or terrestrial origin) are also reported.

Water chemistry parameters		Interface	Lake	Lake ice	Outlet
Nutrients	TP (µg/L)	84.994	5.87	-	6.7
	TN (mg/L)	0.059	0.068	-	0.103
	NO_x (mg/L)	0.047	0.055	-	0.051
	DOC (mg/L)	0.04	0.026	-	0
Pigments (µg/L)	Chlorophylls	0.234	0.162	0.014	0.026
	Carotenes	0.002	0.001	0	0
	Xanthophylls	0.053	0.073	0.003	0.01
Fatty acids (µg/mg)	Algal	0.019	0.144	-	0.14
	Bacterial	0.095	0.037	-	0.034
	Terrestrial	0.029	0.011	-	0.021

Supplementary Table S3. Sequence processing throughout analytical pipeline. DNA concentration (ng/µL), sequencing yield (read count) and DADA2 processing (counts), including the number of filtered reads (truncLen=275,250; maxN=0; maxEE=2,2; truncQ=2), forward (F) and reverse (R) denoised reads, number of merged reads and non-chimeric reads (method “consensus”). These remaining reads are ASVs. Lines in gray show low-yield samples that were eliminated from downstream analyses.

Sample ID	DNA (ng/µL)	Sequencing yield (counts)	DADA2 pipeline (counts)				
			filtered	denoisedF	denoisedR	merged	nonchim (ASV)
CG2GA	0.79	255 430	185 739	180 408	180 842	165 768	117 583
CG2RJA	<LD	1264	414	262	255	234	234
CGCA	0.49	343 738	249 096	245 599	245 996	232 505	177 958
CGST40B	4.9	315 912	236 446	227 476	229 193	205 807	132 959
CGST5A	4.9	277 729	202 111	193 407	195 387	173 521	111 707
CGSTI7B	4.1	380 211	284 182	272 748	275 263	244 890	156 920
CGSTIB	2.7	316 401	233 531	224 996	226 578	209 883	180 828
CGSTOC	3.2	332 835	242 701	226 162	228 900	199 961	135 747
CGT15B	1.53	355 797	266 915	260 270	261 330	235 946	146 094
CGT2JA	0.90	3 384	533	385	391	274	274
CGT5A	1.01	326 957	234 277	228 612	229 909	211 207	145 876
CGTDIA	0.41	238 197	174 285	170 710	170 731	160 975	141 448
CGTGCB	1.44	377 796	287 634	285 204	285 682	272 862	200 611
CGTIBA	<LD	307 600	223 260	218 927	220 060	204 618	160 221
CGTIC	0.51	939	447	292	310	279	279
CGTOA	13.40	13 548	5 959	5 433	5 450	5 243	5222
CGTSA	0.77	334 707	241 671	239 921	239 826	223 184	158331
CGTWFB	0.59	309 262	231 619	226 590	227 117	213 273	158486
CGTWTB	<LD	5425	2 657	1 784	1 826	1 571	1568

Supplementary Table S4. *Polaromonas* 16S genes and gene fragments collected from NCBI using habitat keywords and *taxid* 52972. These genes were aligned, trimmed and combined with ASVs from this study, to compare *Polaromonas* phylogeny across habitats. Published references (Ref) when available are reported in Supplementary references.

Accession #	Sample origin	Sample description	Taxonomy	Sequence reference	Published reference
MT890199.1	Antarctica	Green snow, coastal area of Eastern	Polaromonas sp. strain G.S.42	Smirnova et al. 2020	
NR_25958.1	Antarctica	ATCC:51984	Polaromonas vacuolata strain 34-P	Irgens et al. 1994	
KY405986.1	Antarctica	soil sample from Jarine Peak, King George Island	Polaromonas sp. strain ANT J31B	Romaniukn et al. 2016	
KY405985.1	Antarctica	soil sample from Jarine Peak, King George Island	Polaromonas sp. strain ANT J29B		
KY405981.1	Antarctica	soil sample from Jarine Peak, King George Island	Polaromonas sp. strain ANT J24B		
KY405980.1	Antarctica	soil sample from Jarine Peak, King George Island	Polaromonas sp. strain ANT J23B		
KY405976.1	Antarctica	soil sample from Jarine Peak, King George Island	Polaromonas sp. strain ANT J19B		
KY405972.1	Antarctica	soil sample from Jarine Peak, King George Island	Polaromonas sp. strain ANT J14B		
MG098815.1	Antarctica	Ecology glacier, surface	Polaromonas sp. strain E26S	Ciok et al. 2017	
MG098814.1	Antarctica	Ecology glacier, surface	Polaromonas sp. strain E25S		
MG098813.1	Antarctica	Ecology glacier, cryoconite	Polaromonas sp. strain E24S		
MG098812.1	Antarctica	Ecology glacier, cryoconite	Polaromonas sp. strain E23S		
MG098811.1	Antarctica	Ecology glacier, cryoconite	Polaromonas sp. strain E22S		
MG098810.1	Antarctica	Baranowski glacier, cryoconite	Polaromonas sp. strain B4S		
MG098809.1	Antarctica	Baranowski glacier, cryoconite	Polaromonas sp. strain B3S		
MG098808.1	Antarctica	Baranowski glacier, cryoconite	Polaromonas sp. strain B2S		
MG098807.1	Antarctica	Baranowski glacier, cryoconite	Polaromonas sp. strain B1S		
KC433628.1	Antarctica	na	Polaromonas sp. R3.2	See-too et al. 2013	
KC433619.1	Antarctica	na	Polaromonas sp. L19.9		
KT965159.1	Antarctica	Victorial Valley	Polaromonas sp. B23	Lo Guidice 2015	
KU586646.1	Antarctica	Ecology glacier, surface	Polaromonas sp. E9S	Gawor et al. 2016	Gawor, J. *et al.* Evidence of adaptation, niche separation and microevolution within the genus *Polaromonas* on Arctic and Antarctic glacial surfaces. *Extremophiles* **20**, 403–413 (2016).
KU586645.1	Antarctica	Ecology glacier, surface	Polaromonas sp. E8S		
KU586644.1	Antarctica	Ecology glacier, surface	Polaromonas sp. E7S		
KU586643.1	Antarctica	Ecology glacier, surface	Polaromonas sp. E6S		
KU586642.1	Antarctica	Ecology glacier, surface	Polaromonas sp. E5S		
KU586641.1	Antarctica	Ecology glacier, surface	Polaromonas sp. E3S		
KU586640.1	Antarctica	Ecology glacier, surface	Polaromonas sp. E2S		
KU586639.1	Antarctica	Ecology glacier, surface	Polaromonas sp. E21S		
KU586638.1	Antarctica	Ecology glacier, surface	Polaromonas sp. E20S		
KU586637.1	Antarctica	Ecology glacier, surface	Polaromonas sp. E19S		
KU586636.1	Antarctica	Ecology glacier, surface	Polaromonas sp. E18S		
KU586635.1	Antarctica	Ecology glacier, surface	Polaromonas sp. E17S		
KU586634.1	Antarctica	Ecology glacier, surface	Polaromonas sp. E16S		
KU586633.1	Antarctica	Ecology glacier, surface	Polaromonas sp. E15S		
KU586632.1	Antarctica	Ecology glacier, surface	Polaromonas sp. E14S		
KU586631.1	Antarctica	Ecology glacier, surface	Polaromonas sp. E13S		
KU586630.1	Antarctica	Ecology glacier, surface	Polaromonas sp. E12S		
KU586629.1	Antarctica	Ecology glacier, surface	Polaromonas sp. E11S		
KU586628.1	Antarctica	Ecology glacier, surface	Polaromonas sp. E10S		
GU000429.1	Antarctica	lake sediment	Uncultured Polaromonas sp. clone L6B-55	Shivaji et al. 2011	Shivaji, S. *et al.* Vertical distribution of bacteria in a lake sediment from Antarctica by culture-

					independent and culture-dependent approaches. *Res. Microbiol.* **162**, 191–203 (2011).
KP012213.1	Antarctica	glacier ice	Uncultured Polaromonas sp. clone BMAC-211	Cid et al. 2024	
KP012180.1	Antarctica	glacier ice	Uncultured Polaromonas sp. clone BMAC-108		
KF928875.1	Antarctica	Lake Limnopolar	Uncultured Polaromonas sp. clone H11	Lo Guidice & Michaud 2013	
KF928873.1	Antarctica	Lake Limnopolar	Uncultured Polaromonas sp. clone H05		
KF928870.1	Antarctica	Lake Limnopolar	Uncultured Polaromonas sp. clone G05		
KF928868.1	Antarctica	Lake Limnopolar	Uncultured Polaromonas sp. clone G01		
KF928862.1	Antarctica	Lake Limnopolar	Uncultured Polaromonas sp. clone D07		
KF928845.1	Antarctica	Lake Limnopolar	Uncultured Polaromonas sp. clone A01		
JF728953.1	Antarctica	Ecology glacier, surface	Polaromonas sp. DAB 10Ecl	Zdanowski et al. 2013	Zdanowski, M. K. *et al.* Culturable bacteria community development in postglacial soils of Ecology Glacier, King George Island, Antarctica. *Polar Biol.* **36**, 511–527 (2013).
JF728927.1	Antarctica	Ecology glacier, surface	Polaromonas sp. DAB 7Ecl		
JX196642.1	Antarctica	soil	Polaromonas sp. SON-313 16S	Xiong & Yang 2012	
JQ229549.1	Antarctica	Crater Cirque Lake	Uncultured Polaromonas sp. clone ALC-INI-B3	Michaud et al. 2012	Michaud, L. *et al.* Predominance of *Flavobacterium*, *Pseudomonas*, and *Polaromonas* within the prokaryotic community of freshwater shallow lakes in the northern Victoria Land, East Antarctica. *FEMS Microbiol. Ecol.* **82**, 391–404 (2012).
JQ229547.1	Antarctica	Inexpressible Island Lake 1	Uncultured Polaromonas sp. clone ALC-CC-C1		
FR691432.1	Antarctica	Transantarctic Mountains, Folidas	Polaromonas sp. R-37550	Peeters et al. 2011	Peeters, K., Hodgson, D. A., Convey, P. & Willems, A. Culturable diversity of heterotrophic bacteria in Forlidas Pond (Pensacola Mountains) and Lundström Lake (Shackleton Range), Antarctica. *Microb. Ecol.* **62**, 399–413 (2011).
FR691431.1	Antarctica	Transantarctic Mountains, Folidas	Polaromonas sp. R-38520		
FR691430.1	Antarctica	Transantarctic Mountains, Folidas	Polaromonas sp. R-36732		
FR682711.1	Antarctica	Utsteinen, Sor Rondane Mts, Dronning	Polaromonas sp. R-36500		
FR682710.1	Antarctica	Utsteinen, Sor Rondane Mts, Dronning	Polaromonas sp. R-40127		
U14585.1	Antarctica	Sea ice and water	Polaromonas vacuolata	Gosink & Staley 1995	Irgens, R. L., Gosink, J. J. & Staley, J. T. *Polaromonas vacuolata* gen. nov., sp. nov., a psychrophilic, marine, gas vacuolate bacterium from Antarctica. *International Journal of Systematic Bacteriology* **46**, 822–826 (1996).
MT309509.1	Svalbard	na	Polaromonas glacialis strain 4 VB Sed T8	Thomas & Krushnan 2020	
MN080217.1	Svalbard	na	Polaromonas cryoconiti strain 4 BR2 Sed T5		
MN080216.1	Svalbard	na	Polaromonas eurypsychrophila strain 4 BR2 Sed T2		
MH482339.1	Svalbard	na	Polaromonas eurypsychrophila strain 20BR6T3		
MH482338.1	Svalbard	na	Polaromonas eurypsychrophila strain 20BR6T2		
MH482337.1	Svalbard	na	Polaromonas eurypsychrophila strain 20BR6R17		
MH482329.1	Svalbard	na	Polaromonas eurypsychrophila strain 20ABR13		
MH482325.1	Svalbard	na	Polaromonas eurypsychrophila strain 20ABL8		
MH482320.1	Svalbard	na	Polaromonas eurypsychrophila strain 20ABT4		
MH482319.1	Svalbard	na	Polaromonas eurypsychrophila strain 20ABT8		
MH482318.1	Svalbard	na	Polaromonas eurypsychrophila strain 20ABT6		
MH482315.1	Svalbard	na	Polaromonas eurypsychrophila strain 20ABR10		
MH482314.1	Svalbard	na	Polaromonas eurypsychrophila strain 20ABR6		
MH482291.1	Svalbard	na	Polaromonas eurypsychrophila strain 20BR6R19		
MH482244.1	Svalbard	na	Polaromonas eurypsychrophila strain 4ABT17		
MH482239.1	Svalbard	na	Polaromonas glacialis strain 4ABT2		
MH482231.1	Svalbard	na	Polaromonas eurypsychrophila strain 4ABR7		

MH482227.1	Svalbard	na	Polaromonas glacialis strain 4ABL13		
MG098817.1	Svalbard	Werenskiold glacier cryoconite	Polaromonas sp. strain W14N	Ciok et al. 2017	
MG098816.1	Svalbard	Werenskiold glacier cryoconite	Polaromonas sp. strain W13N		
KU671199.1	China	na	Polaromonas sp.MT-G-S3	Lio & Zhang 2016	
KU586670.1	Svalbard	Werenskiold glacier surface	Polaromonas sp. W9N	Gawor et al. 2016	Gawor, J. *et al.* Evidence of adaptation, niche separation and microevolution within the genus *Polaromonas* on Arctic and Antarctic glacial surfaces. *Extremophiles* **20**, 403–413 (2016).
KU586669.1	Svalbard	Werenskiold glacier surface	Polaromonas sp. W8N		
KU586668.1	Svalbard	Werenskiold glacier surface	Polaromonas sp. W7N		
KU586667.1	Svalbard	Werenskiold glacier surface	Polaromonas sp. W6N		
KU586666.1	Svalbard	Werenskiold glacier surface	Polaromonas sp. W5N		
KU586665.1	Svalbard	Werenskiold glacier surface	Polaromonas sp. W4N		
KU586664.1	Svalbard	Werenskiold glacier surface	Polaromonas sp. W3N		
KU586663.1	Svalbard	Werenskiold glacier surface	Polaromonas sp. W2N		
KU586662.1	Svalbard	Werenskiold glacier surface	Polaromonas sp. W1N		
KU586661.1	Svalbard	Werenskiold glacier surface	Polaromonas sp. W12N		
KU586660.1	Svalbard	Werenskiold glacier surface	Polaromonas sp. W11N		
KU586659.1	Svalbard	Werenskiold glacier surface	Polaromonas sp. W10N		
KU586658.1	Svalbard	Hans glacer, surface	Polaromonas sp. H9N		
KU586657.1	Svalbard	Hans glacer, surface	Polaromonas sp. H8N		
KU586656.1	Svalbard	Hans glacer, surface	Polaromonas sp. H7N		
KU586655.1	Svalbard	Hans glacer, surface	Polaromonas sp. H6N		
KU586654.1	Svalbard	Hans glacer, surface	Polaromonas sp. H5N		
KU586653.1	Svalbard	Hans glacer, surface	Polaromonas sp. H4N		
KU586652.1	Svalbard	Hans glacer, surface	Polaromonas sp. H3N		
KU586651.1	Svalbard	Hans glacer, surface	Polaromonas sp. H2N		
KU586650.1	Svalbard	Hans glacer, surface	Polaromonas sp. H1N		
KU586649.1	Svalbard	Hans glacer, surface	Polaromonas sp. H12N		
KU586648.1	Svalbard	Hans glacer, surface	Polaromonas sp. H11N		
KU586647.1	Svalbard	Hans glacer, surface	Polaromonas sp. H10N		
JX205214.1	Svalbard	glacier cryoconite, Ny-Alesund	Polaromonas jejuensis strain MLB-45	Singh et al. 2012	Singh, P., Singh, S. M. & Dhakephalkar, P. Diversity, cold active enzymes and adaptation strategies of bacteria inhabiting glacier cryoconite holes of High Arctic. *Extremophiles* **18**, 229–242 (2014).
JX205211.1	Svalbard	glacier cryoconite, Ny-Alesund	Polaromonas naphthalenivorans strain MLB-44		
HQ622745.1	Svalbard	Austre Lovenbreen glacier ice core, Ny-Alesund	Uncultured Polaromonas sp. clone IC4073	Zeng et al. 2010	
HQ595202.1	Svalbard	Austre Lovenbreen glacier ice core, Ny-Alesund	Uncultured Polaromonas sp. clone IC3028		
AM940813.1	Svalbard	Midtre Lovenbreen glacier moraine, Ny-Alesund	Uncultured Polaromonas sp.	Borin et al. 2008	
AM940793.1	Svalbard	Midtre Lovenbreen glacier moraine, Ny-Alesund	Uncultured Polaromonas sp.		
AM940792.1	Svalbard	Midtre Lovenbreen glacier moraine, Ny-Alesund	Uncultured Polaromonas sp.		
AM940542.1	Svalbard	Midtre Lovenbreen glacier moraine, Ny-Alesund	Uncultured Polaromonas sp.		
AM940524.1	Svalbard	Midtre Lovenbreen glacier moraine, Ny-Alesund	Uncultured Polaromonas sp.		
FJ946540.1	Svalbard	meltwater	Uncultured Polaromonas sp. clone SSIM-F2v	Larose et al. 2009	Larose, C. *et al.* Microbial sequences retrieved from environmental samples from seasonal Arctic snow and meltwater from Svalbard, Norway. *Extremophiles* **14**, 205–212 (2010).
FJ946536.1	Svalbard	meltwater	Uncultured Polaromonas sp. clone SSIM-D10n		
FJ946531.1	Svalbard	meltwater	Uncultured Polaromonas sp. clone SSIM-C12n		
FJ946515.1	Svalbard	snow	Uncultured Polaromonas sp. clone LSS-C2		
FM955859.1	Svalbard	sediment, Midtre Lovenbreen glacier	Polaromonas sp. Asd M3-1	Vardan Reddy et al. 2008	Vardhan Reddy, P. V. *et al.* Bacterial diversity and bioprospecting for cold-active enzymes from culturable bacteria associated with sediment from a melt water stream of Midtre Loenbreen glacier,

					an Arctic glacier. *Res. Microbiol.* **160**, 538–546 (2009).
JX950032.1	China	glacier	Polaromonas sp. TMT2-15-4	Liu et al. 2012	Liu, Q., Tian, J.-H., Liu, H.-C., Zhou, Y.-G. & Xin, Y.-H. *Cryobacterium ruanii* sp. nov. and *Cryobacterium breve* sp. nov., isolated from glaciers. *Int. J. Syst. Evol. Microbiol.* **70**, 1918–1923 (2020).
JX950031.1	China	glacier	Polaromonas sp. TMT1-48		
JX950030.1	China	glacier	Polaromonas sp. TMB1-4		
JX950029.1	China	glacier	Polaromonas sp. TMT2-13-2		
JX950028.1	China	glacier	Polaromonas sp. TMN-26		
JX950027.1	China	glacier	Polaromonas sp. TMB1-23		
JX950026.1	China	glacier	Polaromonas sp. TMT4-24		
JX950025.1	China	glacier	Polaromonas sp. TMT4-21		
JX950024.1	China	glacier	Polaromonas sp. TMT2-58		
JX950023.1	China	glacier	Polaromonas sp. TMT2-31-2		
JX950022.1	China	glacier	Polaromonas sp. TMT2-30-2		
JX950021.1	China	glacier	Polaromonas sp. TMT2-19		
JX950020.1	China	glacier	Polaromonas sp. TMT2-3-2		
JX950019.1	China	glacier	Polaromonas sp. TMT1-54		
JX950018.1	China	glacier	Polaromonas sp. TMT1-26-2		
JX950017.1	China	glacier	Polaromonas sp. TMT1-44		
JX950016.1	China	glacier	Polaromonas sp. TMT1-35		
JX950015.1	China	glacier	Polaromonas sp. TMT1-41		
JX950014.1	China	glacier	Polaromonas sp. TmT2-46		
JX950013.1	China	glacier	Polaromonas sp. TmT2-45-1		
JX950012.1	China	glacier	Polaromonas sp. TmT2-44		
JX950011.1	China	glacier	Polaromonas sp. TmT2-41		
JX950010.1	China	glacier	Polaromonas sp. TmT2-40-1		
JX950009.1	China	glacier	Polaromonas sp. TmT2-32		
JX950008.1	China	glacier	Polaromonas sp. TmT2-18-1		
JX950007.1	China	glacier	Polaromonas sp. TmT2-17-2		
JX949215.1	China	glacier	Polaromonas sp. Sr67		
JX949214.1	China	glacier	Polaromonas sp. Sr52		
JX949213.1	China	glacier	Polaromonas sp. Sr42		
JX949212.1	China	glacier	Polaromonas sp. Sr41		
JX949211.1	China	glacier	Polaromonas sp. Sr34		
JX949210.1	China	glacier	Polaromonas sp. Sr23		
JX949209.1	China	glacier	Polaromonas sp. Sr4		
JX949208.1	China	glacier	Polaromonas sp. Ht10		
NR_109013.1	Austria	Tyrol, Oetztaler Alps, Pitztaler Joechl cryoconite	Polaromonas glacialis strain Cr4-12	Margesin et al. 2010	Margesin, R., Spröer, C., Zhang, D.-C. & Busse, H.-J. *Polaromonas glacialis* sp. nov. and *Polaromonas cryoconiti* sp. nov., isolated from alpine glacier cryoconite. *Int. J. Syst. Evol. Microbiol.* **62**, 2662–2668 (2012).
NR_109012.1	Austria	Tyrol, Oetztaler Alps, Pitztaler Joechl cryoconite	Polaromonas cryoconiti strain Cr4-35		
MG973003.1	India	Chhota Shigri glacier cryoconite sediment	Polaromonas glacialis strain S7A-3	Naik et al. 2018	
MG973002.1	India	Chhota Shigri glacier cryoconite sediment	Polaromonas glacialis strain S7A-15		
MF555703.1	India	Himalaya glacier cryoconite	Polaromonas glacialis strain SD-20	Singh 2017	
MH174178.1	China	Tianshan no1 glacier suglacial sediment	Uncultured Polaromonas sp. clone TSJW 97	Qi 2018	
MH174173.1	China	Tianshan no1 glacier suglacial sediment	Uncultured Polaromonas sp. clone TSD 16		
MH174158.1	China	Tianshan no1 glacier suglacial sediment	Uncultured Polaromonas sp. clone TSX 64		
MH174157.1	China	Tianshan no1 glacier suglacial sediment	Uncultured Polaromonas sp. clone TSFC 40		

MH174147.1	China	Tianshan no1 glacier suglacial sediment	Uncultured Polaromonas sp. clone TSFC 64		
MH174134.1	China	Tianshan no1 glacier suglacial sediment	Uncultured Polaromonas sp. clone TSX 114		
MH174123.1	China	Tianshan no1 glacier suglacial sediment	Uncultured Polaromonas sp. clone TSD 199		
MH174120.1	China	Tianshan no1 glacier suglacial sediment	Uncultured Polaromonas sp. clone TSX 59		
MH174118.1	China	Tianshan no1 glacier suglacial sediment	Uncultured Polaromonas sp. clone TSD 115		
MH174109.1	China	Tianshan no1 glacier suglacial sediment	Uncultured Polaromonas sp. clone TSD 52		
MH174103.1	China	Tianshan no1 glacier suglacial sediment	Uncultured Polaromonas sp. clone TSD 101		
MH174100.1	China	Tianshan no1 glacier suglacial sediment	Uncultured Polaromonas sp. clone TSX 1		
MH174098.1	China	Tianshan no1 glacier suglacial sediment	Uncultured Polaromonas sp. clone TSX 102		
MH174097.1	China	Tianshan no1 glacier suglacial sediment	Uncultured Polaromonas sp. clone TSD 118		
MH174093.1	China	Tianshan no1 glacier suglacial sediment	Uncultured Polaromonas sp. clone TSX 61		
MG952607.1	India	Patsio glacier cyroconite sediment	Polaromonas glacialis strain BMP50	Rathore et al. 2018	
MG952605.1	India	Patsio glaciercyroconite sediment	Polaromonas glacialis strain S4PB45		
MG952601.1	India	Patsio glacier cryoconite water	Polaromonas glacialis strain S4PB32		
MG952600.1	India	Patsio crycoconite	Polaromonas glacialis strain S3PB51		
MG952599.1	India	Patsio glacier cryoconite water	Polaromonas glacialis strain S4PB2		
MG952598.1	India	Patsio glacier cryoconite water	Polaromonas glacialis strain S4PB14		
MG952597.1	India	Patsio glacier cryoconite water	Polaromonas glacialis strain S4PB52		
MG952587.1	India	Patsio glacier cryoconite water	Polaromonas glacialis strain PM34		
JX949585.1	China	glacier	Polaromonas sp. MDB2-14	Liu & Xin 2012	
AB991680.1	Alaska	ice worm microbiome	Polaromonas sp. MsC-08-1CB1-02	Murakami & Hongoh 2014	Murakami, T. *et al.* Census of bacterial microbiota associated with the glacier ice worm *Mesenchytraeus solifugus*. *FEMS Microbiol. Ecol.* **91**, (2015).
GQ421106.1	India	Roopkund glacier soil	Uncultured Polaromonas sp. clone RUGL1-204	Shivaji et al. 2009	Pradhan, S. *et al.* Bacterial biodiversity from Roopkund Glacier, Himalayan mountain ranges, India. *Extremophiles* **14**, 377–395 (2010).
GQ421095.1	India	Roopkund glacier soil	Uncultured Polaromonas sp. clone RUGL1-169		
GQ421051.1	India	Roopkund glacier soil	Uncultured Polaromonas sp. clone RUGL1-292		
GQ421025.1	India	Roopkund glacier soil	Uncultured Polaromonas sp. clone RUGL1-93		
GQ420963.1	India	Roopkund glacier soil	Uncultured Polaromonas sp. clone RUGL1-538		
GQ420917.1	India	Roopkund glacier soil	Uncultured Polaromonas sp. clone RUGL1-428		
KP724795.1	Antarctica	South Shetland glacier ice	Uncultured Polaromonas sp. clone BECO-27	Cid et al. 2015	
KP724771.1	Antarctica	South Shetland glacier ice	Uncultured Polaromonas sp. clone BJOH-155		
KP724764.1	Antarctica	South Shetland glacier ice	Uncultured Polaromonas sp. clone BJOH-141		
KP724763.1	Antarctica	South Shetland glacier ice	Uncultured Polaromonas sp. clone BJOH-137		
KP724754.1	Antarctica	South Shetland glacier ice	Uncultured Polaromonas sp. clone BJOH-119		
KP724750.1	Antarctica	South Shetland glacier ice	Uncultured Polaromonas sp. clone BJOH-111		
KP724739.1	Antarctica	South Shetland glacier ice	Uncultured Polaromonas sp. clone BJOH-89		
KP724738.1	Antarctica	South Shetland glacier ice	Uncultured Polaromonas sp. clone BJOH-88		
KP724720.1	Antarctica	South Shetland glacier ice	Uncultured Polaromonas sp. clone BJOH-50		
KP724703.1	Antarctica	South Shetland glacier ice	Uncultured Polaromonas sp. clone BJOH-14		
HM583568.2	Austria	Tyrol, Oetztaler Alps, Pitztaler Joechl cryoconite	Polaromonas glacialis strain Cr4-12	Margesin et al. 2010	Margesin, R., Spröer, C., Zhang, D.-C. & Busse, H.-J. *Polaromonas glacialis* sp. nov. and *Polaromonas cryoconiti* sp. nov., isolated from alpine glacier cryoconite. *Int. J. Syst. Evol. Microbiol.* **62**, 2662–2668 (2012).
HM583567.2	Austria	Tyrol, Oetztaler Alps, Pitztaler Joechl cryoconite	Polaromonas cryoconiti strain Cr4-35		
EU809904.1	India	Himalayan proglacial soil	Uncultured Polaromonas sp. clone KS-160	Srinivas et al. 2011	Srinivas, T. N. R. *et al.* Comparison of bacterial diversity in proglacial soil from Kafni Glacier, Himalayan Mountain ranges, India, with the
EU809727.1	India	Himalayan proglacial soil	Uncultured Polaromonas sp. clone KS-476		
EU809515.1	India	Himalayan proglacial soil	Uncultured Polaromonas sp. clone KS-408		

					bacterial diversity of other glaciers in the world. *Extremophiles* **15**, 673–690 (2011).
GQ287523.1	India	Pinadri glacier soil	Uncultured Polaromonas sp. clone P1s-302	Shivaji et al. 2009	Shivaji, S. *et al.* Bacterial diversity of soil in the vicinity of Pindari glacier, Himalayan mountain ranges, India, using culturable bacteria and soil 16S rRNA gene clones. *Extremophiles* **15**, 1–22 (2011).
HM156127.1	China	glacier foield soil	Polaromonas sp. TSE17	Yue et al. 2010	
GQ366620.1	India	Roopkund glacier soil	Uncultured Polaromonas sp. clone RUGL6-368	Shivaji et al. 2009	Shivaji, S. *et al.* Bacterial diversity of soil in the vicinity of Pindari glacier, Himalayan mountain ranges, India, using culturable bacteria and soil 16S rRNA gene clones. *Extremophiles* **15**, 1–22 (2011).
GQ366530.1	India	Roopkund glacier soil	Uncultured Polaromonas sp. clone RUGL6-213		
GQ366469.1	India	Roopkund glacier soil	Uncultured Polaromonas sp. clone RUGL6-125		
GQ366441.1	India	Roopkund glacier soil	Uncultured Polaromonas sp. clone RUGL6-86		
GU213397.1	Switzerland	Damma glacier granite sand	Polaromonas sp. 190	Lapanje et al. 2009	
FJ979859.1	China	Tianshan No1 glacier snow	Polaromonas sp. tsz31	Zhang et al. 2009	
FJ979854.1	China	Tianshan No1 glacier snow	Polaromonas sp. tsz24		
EF423340.1	China	Tianshan No1 glacier snow	Polaromonas sp. 3010	Wang et al 2007	
EF423333.1	China	Tianshan No1 glacier snow	Polaromonas sp. 1024		
EF423330.1	China	Tianshan No1 glacier snow	Polaromonas sp. 1020		
EF423325.1	China	Tianshan No1 glacier snow	Polaromonas sp. 1011		
EF423322.1	China	Tianshan No1 glacier snow	Polaromonas sp. 1006		
MK670532.1	Svalbard	subglacial ice	Polaromonas sp. strain EXB-L-2565	Perini et al. 2019	Perini, L., Gostinčar, C. & Gunde-Cimerman, N. Fungal and bacterial diversity of Svalbard subglacial ice. *Sci. Rep.* **9**, 20230 (2019).
MK670510.1	Svalbard	subglacial ice	Polaromonas sp. strain EXB-L-2558		
NR_149767.1	China	ice core	149767.1 Polaromonas eurypsychrophila strain	Xing et al. 2014	Xing, T. *et al. Polaromonas eurypsychrophila* sp. nov., isolated from an ice core. *Int. J. Syst. Evol. Microbiol.* **66**, 2497–2501 (2016).
KP013181.1	China	ice core	Polaromonas eurypsychrophila strain B717-2		
KU179860.1	Canada	sea-ice melt pool	Polaromonas hydrogenivorans strain RKAT288	Thomas, 2015	
KU179859.1	Canada	sea-ice melt pool	Polaromonas glacialis strain RKAT094		
KM873052.1	Tibet	Ulugh Muztagh ice core	Polaromonas sp. B898-3	Guo 2014	
KF295827.1	Tibet	Zuoquipu ice core	Polaromonas sp. Z96	Liu 2013	
KF295443.1	Tibet	Zuoquipu ice core	Polaromonas sp. Z113		
KF295441.1	Tibet	Zuoquipu ice core	Polaromonas sp. Z106		
KF295440.1	Tibet	Zuoquipu ice core	Polaromonas sp. Z104		
KF295051.1	Tibet	Ningjingangsang ice core	Polaromonas sp. N4 99 4		
KF295050.1	Tibet	Ningjingangsang ice core	Polaromonas sp. N4 99 3		
KF295043.1	Tibet	Ningjingangsang ice core	Polaromonas sp. N4 98 5		
KF295040.1	Tibet	Ningjingangsang ice core	Polaromonas sp. N4 98 2		
KF295024.1	Tibet	Ningjingangsang ice core	Polaromonas sp. N4 94 3		
HQ144221.1	Greenland	basal ice	Polaromonas sp. Bis 20	Finster 2010	
FJ477327.1	Antarctica	Kamb ice stream	Uncultured bacterium clone A	Lanoil et al. 2008	Lanoil, B. *et al.* Bacteria beneath the West Antarctic Ice Sheet. *Environ. Microbiol.* **11**, 609–615 (2009).
FJ979854.1	China	Glacier no 1	Polaromonas sp. tsz24	Zhang et al. 2009	
FJ979859.1	China	Glacier no 1	Polaromonas sp. tsz31		
EF423322.1	China	Glacier no 1	Polaromonas sp. 1006		
EF423325.1	China	Glacier no 1	Polaromonas sp. 1011		
EF423330.1	China	Glacier no 1	Polaromonas sp. 1020		
EF423333.1	China	Glacier no 1	Polaromonas sp. 1024		

EF423340.1	China	Glacier no 1	Polaromonas sp. 3010		
EU978852.1	Germany	Schneeferner glacier	ice bacterium sp. glbI11	Simon et al. 2008	Simon, C., Wiezer, A., Strittmatter, A. W. & Daniel, R. Phylogenetic diversity and metabolic potential revealed in a glacier ice metagenome. *Appl. Environ. Microbiol.* **75**, 7519–7526 (2009).
EU636024.1	Antarctica	Collins glacier	bacterium G076	Garcia Echauri et al. 2008	García-Echauri, S. A., Gidekel, M., Gutiérrez-Moraga, A., Santos, L. & De León-Rodríguez, A. Isolation and phylogenetic classification of culturable psychrophilic prokaryotes from the Collins glacier in the Antarctica. *Folia Microbiol.* **56**, 209–214 (2011).
EU636026.1	Antarctica	Collins glacier	bacterium G024		
EU636027.1	Antarctica	Collins glacier	bacterium G020		
EU636029.1	Antarctica	Collins glacier	bacterium G088		
AY315174.1	New Zealand	Franz Josef glacier	Glacier bacterium FJI50	Foght et al. 2003	Foght, J. *et al.* Culturable bacteria in subglacial sediments and ice from two Southern hemisphere glaciers. *Microb. Ecol.* **47**, (2004).
AY315175.1	New Zealand	Franz Josef glacier	Glacier bacterium FJS17		
AY315178.1	New Zealand	Franz Josef glacier	Glacier bacterium FJS31		
AY315176.1	New Zealand	Fox glacier	Glacier bacterium FXS33		
AY315177.1	New Zealand	Fox glacier	Glacier bacterium FXS1		
DQ628933.1	Canada	John Evans glacier	Uncultured Comamonadaceae bacterium clone SOC A20(17)	Cheng & Foght 2006	Cheng, S. M. & Foght, J. M. Cultivation-independent and -dependent characterization of Bacteria resident beneath John Evans Glacier: characterization of subglacial bacterial communities. *FEMS Microbiol. Ecol.* **59**, 318–330 (2007).
DQ628932.1	Canada	John Evans glacier	Uncultured Comamonadaceae bacterium clone SOC A4(5)		
DQ628934.1	Canada	John Evans glacier	Uncultured Comamonadaceae bacterium clone SIB2 1B		
DQ628935.1	Canada	John Evans glacier	Uncultured Comamonadaceae bacterium clone BFM 6B		
DQ628936.1	Canada	John Evans glacier	Uncultured Comamonadaceae bacterium clone BF M20(8)		
DQ628937.1	Canada	John Evans glacier	Uncultured Comamonadaceae bacterium clone A20(82) 16S		
DQ628938.1	Canada	John Evans glacier	Uncultured Comamonadaceae bacterium clone SIB2 1G		
DQ628939.1	Canada	John Evans glacier	Uncultured Comamonadaceae bacterium clone SOC1 1C		
DQ628940.1	Canada	John Evans glacier	Uncultured Comamonadaceae bacterium clone SOC1 1B		
DQ530258.1	Canada	John Evans glacier	Uncultured Comamonadaceae bacterium clone BFA H1		
DQ228409.1	Canada	John Evans glacier	Uncultured bacterium clone JEG.g5		
DQ228403.1	Canada	John Evans glacier	Uncultured bacterium clone JEG.e1		
GQ306091.1	Peru	Cordillera Vilcanota periglacial soil	bacterium clone 0MHA A11	Schmidt et al. 2009	Schmidt, S. K. *et al.* Microbial activity and diversity during extreme freeze–thaw cycles in periglacial soils, 5400 m elevation, Cordillera Vilcanota, Perú. *Extremophiles* **13**, 807–816 (2009).
JF719323.1	USA (Colorado)	Arikaree glacier	Polaromonas sp. clone T3P2 D02	Darcy et al. 2011	Darcy, J. L., Lynch, R. C., King, A. J., Robeson, M. S. & Schmidt, S. K. Global distribution of *Polaromonas* phylotypes - evidence for a highly successful dispersal capacity. *PLoS ONE* **6**, e23742 (2011).
JF719322.1	USA (Colorado)	Arikaree glacier	Polaromonas sp. clone T4P2 A31		
JF719329.1	USA (Colorado)	Arikaree glacier	Polaromonas sp. clone T5P2 A26		
GQ396863.1	USA (Alaska)	Mendenhall glacier	bacterium clone AK1AB2 06A	Sattin et al. 2009	

GQ396949.1	USA (Alaska)	Mendenhall glacier	bacterium clone AK4AB2 12A	Sattin, S. R. *et al.* Functional shifts in unvegetated, perhumid, recently-deglaciated soils do not correlate with shifts in soil bacterial community composition. *J. Microbiol.* **47**, 673–681 (2009).
GQ396971.1	USA (Alaska)	Mendenhall glacier	bacterium clone AK1DE1 04B	
JF719324.1	USA (Alaska)	Toklat glacier	Polaromonas sp. clone N21B05H	
JF719325.1	USA (Alaska)	Toklat glacier	Polaromonas sp. clone NG 130 01D	
JF719326.1	USA (Alaska)	Toklat glacier	Polaromonas sp. clone NG 130 03D	
JF719327.1	USA (Alaska)	Toklat glacier	Polaromonas sp. clone NG 130 06B	
JF7193278.1	USA (Alaska)	Toklat glacier	Polaromonas sp. clone NG 137 08C	
JF719331.1	USA (Alaska)	Toklat glacier	Polaromonas sp. clone N21A02C	
JF719332.1	USA (Alaska)	Toklat glacier	Polaromonas sp. clone N22A02B	
JF719333.1	USA (Alaska)	Toklat glacier	Polaromonas sp. clone N28A03H	
JF719334.1	USA (Alaska)	Toklat glacier	Polaromonas sp. clone N28B08E	
JF719335.1	USA (Alaska)	Toklat glacier	Polaromonas sp. clone NG 130 09B	
JF719336.1	USA (Alaska)	Toklat glacier	Polaromonas sp. clone NG 130 09H	
JF719337.1	USA (Alaska)	Toklat glacier	Polaromonas sp. clone NG 134 03E	
JF719338.1	USA (Alaska)	Toklat glacier	Polaromonas sp. clone NG 137 06B	
JF729309.1	USA (Alaska)	Toklat glacier	Polaromonas sp. clone 05B	

Supplementary Table S5. Core microbiome across the Thores glacier-lake-outflow continuum. List of the 141 core taxa identified, where core taxa were defined as ASVs detected in over 50% of samples with a relative abundance greater than 1%.

Phylum	Class	Order	Family	Genus	Species
Verrucomicrobiota	Verrucomicrobiae	Methylacidiphilales	Methylacidiphilaceae	NA	NA
Verrucomicrobiota	Verrucomicrobiae	Verrucomicrobiales	Rubritaleaceae	Luteolibacter	NA
Verrucomicrobiota	Verrucomicrobiae	Opitutales	Opitutaceae	Lacunisphaera	NA
Verrucomicrobiota	Verrucomicrobiae	Opitutales	Opitutaceae	NA	NA
Verrucomicrobiota	Verrucomicrobiae	Pedosphaerales	Pedosphaeraceae	SH3-11	NA
Verrucomicrobiota	Verrucomicrobiae	Pedosphaerales	Pedosphaeraceae	SH3-11	NA
Verrucomicrobiota	Verrucomicrobiae	Pedosphaerales	Pedosphaeraceae	NA	NA
Verrucomicrobiota	Verrucomicrobiae	Pedosphaerales	Pedosphaeraceae	DEV114	NA
Verrucomicrobiota	Verrucomicrobiae	Opitutales	Opitutaceae	Opitutus	NA
Proteobacteria	Gammaproteobacteria	Burkholderiales	Comamonadaceae	Delftia	NA
Proteobacteria	Gammaproteobacteria	Burkholderiales	Comamonadaceae	Polaromonas	NA
Proteobacteria	Gammaproteobacteria	Burkholderiales	Comamonadaceae	Rhodoferax	NA
Proteobacteria	Gammaproteobacteria	Burkholderiales	Oxalobacteraceae	Actimicrobium	NA
Proteobacteria	Gammaproteobacteria	Burkholderiales	Comamonadaceae	Polaromonas	NA
Proteobacteria	Gammaproteobacteria	Burkholderiales	Comamonadaceae	NA	NA
Proteobacteria	Gammaproteobacteria	Burkholderiales	Comamonadaceae	Polaromonas	eurypsychrophila
Proteobacteria	Gammaproteobacteria	Burkholderiales	Comamonadaceae	Limnohabitans	NA
Proteobacteria	Gammaproteobacteria	Burkholderiales	Comamonadaceae	Roseateles	NA
Proteobacteria	Gammaproteobacteria	Burkholderiales	Methylophilaceae	Methylotenera	NA
Proteobacteria	Alphaproteobacteria	Sphingomonadales	Sphingomonadaceae	Polymorphobacter	NA
Proteobacteria	Gammaproteobacteria	Burkholderiales	Comamonadaceae	Rhodoferax	NA
Proteobacteria	Alphaproteobacteria	Sphingomonadales	Sphingomonadaceae	Sphingomonas	NA
Proteobacteria	Gammaproteobacteria	Burkholderiales	Comamonadaceae	Polaromonas	NA
Proteobacteria	Gammaproteobacteria	Burkholderiales	Comamonadaceae	Rhodoferax	NA
Proteobacteria	Gammaproteobacteria	Burkholderiales	Comamonadaceae	NA	NA
Proteobacteria	Gammaproteobacteria	Burkholderiales	Alcaligenaceae	GKS98_freshwater_group	NA
Proteobacteria	Gammaproteobacteria	Burkholderiales	Oxalobacteraceae	Massilia	NA
Proteobacteria	Gammaproteobacteria	Xanthomonadales	Xanthomonadaceae	Arenimonas	NA
Proteobacteria	Gammaproteobacteria	Burkholderiales	Comamonadaceae	NA	NA
Proteobacteria	Gammaproteobacteria	Burkholderiales	Burkholderiaceae	Polynucleobacter	NA
Proteobacteria	Alphaproteobacteria	Acetobacterales	Acetobacteraceae	Rhodovastum	NA
Proteobacteria	Gammaproteobacteria	Pseudomonadales	Spongiibacteraceae	BD1-7_clade	NA
Proteobacteria	Gammaproteobacteria	Burkholderiales	Nitrosomonadaceae	GOUTA6	NA
Proteobacteria	Alphaproteobacteria	Caulobacterales	Caulobacteraceae	Phenylobacterium	NA
Proteobacteria	Gammaproteobacteria	Burkholderiales	TRA3-20	NA	NA
Proteobacteria	Alphaproteobacteria	Acetobacterales	Acetobacteraceae	Roseomonas	arctica
Proteobacteria	Gammaproteobacteria	Burkholderiales	Nitrosomonadaceae	MND1	NA
Proteobacteria	Alphaproteobacteria	Rhodobacterales	Rhodobacteraceae	Pseudorhodobacter	NA
Proteobacteria	Gammaproteobacteria	JG36-TzT-191	NA	NA	NA

Proteobacteria	Gammaproteobacteria	Burkholderiales	Nitrosomonadaceae	966-1	NA
Proteobacteria	Gammaproteobacteria	Burkholderiales	TRA3-20	NA	NA
Proteobacteria	Gammaproteobacteria	Burkholderiales	Comamonadaceae	Rhodoferax	NA
Proteobacteria	Alphaproteobacteria	Acetobacterales	Acetobacteraceae	NA	NA
Proteobacteria	Alphaproteobacteria	Rhodospirillales	Rhodospirillaceae	NA	NA
Proteobacteria	Gammaproteobacteria	Burkholderiales	Sutterellaceae	AAP99	NA
Proteobacteria	Alphaproteobacteria	Sphingomonadales	Sphingomonadaceae	Sandarakinorhabdus	NA
Planctomycetota	Phycisphaerae	Phycisphaerales	Phycisphaeraceae	CL500-3	NA
Planctomycetota	Phycisphaerae	Phycisphaerales	Phycisphaeraceae	CL500-3	NA
Planctomycetota	Planctomycetes	Gemmatales	Gemmataceae	NA	NA
Planctomycetota	Phycisphaerae	Phycisphaerales	Phycisphaeraceae	CL500-3	NA
Planctomycetota	Planctomycetes	Pirellulales	Pirellulaceae	NA	NA
Planctomycetota	Planctomycetes	Gemmatales	Gemmataceae	Gemmata	NA
Planctomycetota	Planctomycetes	Gemmatales	Gemmataceae	NA	NA
Planctomycetota	Planctomycetes	Gemmatales	Gemmataceae	NA	NA
Planctomycetota	Planctomycetes	Pirellulales	Pirellulaceae	NA	NA
Planctomycetota	Planctomycetes	Pirellulales	Pirellulaceae	Pirellula	NA
Planctomycetota	Planctomycetes	Gemmatales	Gemmataceae	Fimbriiglobus	NA
Planctomycetota	Phycisphaerae	Phycisphaerales	Phycisphaeraceae	CL500-3	NA
Planctomycetota	Phycisphaerae	Phycisphaerales	Phycisphaeraceae	CL500-3	NA
Planctomycetota	Planctomycetes	Pirellulales	Pirellulaceae	NA	NA
Planctomycetota	Phycisphaerae	Phycisphaerales	Phycisphaeraceae	CL500-3	NA
Planctomycetota	Planctomycetes	Gemmatales	Gemmataceae	Gemmata	NA
Planctomycetota	Planctomycetes	Planctomycetales	Rubinisphaeraceae	NA	NA
Patescibacteria	Saccharimonadia	Saccharimonadales	NA	NA	NA
Patescibacteria	Saccharimonadia	Saccharimonadales	NA	NA	NA
Patescibacteria	Saccharimonadia	Saccharimonadales	NA	NA	NA
Myxococcota	Polyangia	Blfdi19	NA	NA	NA
Gemmatimonadota	Gemmatimonadetes	Gemmatimonadales	Gemmatimonadaceae	Gemmatimonas	NA
Gemmatimonadota	Gemmatimonadetes	Gemmatimonadales	Gemmatimonadaceae	NA	NA
Gemmatimonadota	Gemmatimonadetes	Gemmatimonadales	Gemmatimonadaceae	NA	NA
Gemmatimonadota	Gemmatimonadetes	Gemmatimonadales	Gemmatimonadaceae	NA	NA
Dependentiae	Babeliae	Babeliales	NA	NA	NA
Deinococcota	Deinococci	Deinococcales	Deinococcaceae	Deinococcus	NA
Cyanobacteria	Cyanobacteriia	Synechococcales	Cyanobiaceae	Cyanobium_PCC-6307	NA
Chloroflexi	Anaerolineae	Anaerolineales	Anaerolineaceae	NA	NA
Bdellovibrionota	Oligoflexia	0319-6G20	NA	NA	NA
Bacteroidota	Bacteroidia	Cytophagales	Hymenobacteraceae	Hymenobacter	frigidus
Bacteroidota	Bacteroidia	Cytophagales	Hymenobacteraceae	Hymenobacter	NA
Bacteroidota	Kapabacteria	Kapabacteriales	NA	NA	NA
Bacteroidota	Bacteroidia	Cytophagales	Spirosomaceae	Arcicella	NA
Bacteroidota	Bacteroidia	Sphingobacteriales	Sphingobacteriaceae	Pedobacter	NA
Bacteroidota	Bacteroidia	Cytophagales	Spirosomaceae	Pseudarcicella	NA
Bacteroidota	Bacteroidia	Cytophagales	Hymenobacteraceae	Hymenobacter	NA
Bacteroidota	Bacteroidia	Chitinophagales	Chitinophagaceae	Dinghuibacter	NA

Bacteroidota	Bacteroidia	Sphingobacteriales	Sphingobacteriaceae	Pedobacter	NA
Bacteroidota	Bacteroidia	Flavobacteriales	Flavobacteriaceae	Flavobacterium	NA
Bacteroidota	Bacteroidia	Chitinophagales	Chitinophagaceae	NA	NA
Bacteroidota	Bacteroidia	Chitinophagales	Chitinophagaceae	Sediminibacterium	NA
Bacteroidota	Bacteroidia	Chitinophagales	Chitinophagaceae	Ferruginibacter	NA
Bacteroidota	Bacteroidia	Sphingobacteriales	env.OPS_17	NA	NA
Bacteroidota	Bacteroidia	Flavobacteriales	Crocinitomicaceae	Fluviicola	NA
Bacteroidota	Bacteroidia	Sphingobacteriales	NS11-12_marine_group	NA	NA
Bacteroidota	Kryptonia	Kryptoniales	BSV26	NA	NA
Bacteroidota	Bacteroidia	Cytophagales	Cyclobacteriaceae	Algoriphagus	NA
Bacteroidota	Bacteroidia	Sphingobacteriales	Sphingobacteriaceae	Pedobacter	luteus
Bacteroidota	Bacteroidia	Chitinophagales	Chitinophagaceae	Sediminibacterium	NA
Bacteroidota	Bacteroidia	Sphingobacteriales	Sphingobacteriaceae	Pedobacter	NA
Bacteroidota	Bacteroidia	Sphingobacteriales	Sphingobacteriaceae	Pedobacter	NA
Bacteroidota	Bacteroidia	Flavobacteriales	Crocinitomicaceae	Fluviicola	NA
Bacteroidota	Bacteroidia	Flavobacteriales	Cryomorphaceae	NA	NA
Bacteroidota	Bacteroidia	Sphingobacteriales	env.OPS_17	NA	NA
Armatimonadota	Armatimonadia	Armatimonadales	Armatimonadaceae	Armatimonas	NA
Actinobacteriota	Actinobacteria	Micrococcales	Microbacteriaceae	Parafrigoribacterium	NA
Actinobacteriota	Actinobacteria	Frankiales	Sporichthyaceae	Candidatus_Planktophila	NA
Actinobacteriota	Actinobacteria	Micrococcales	Microbacteriaceae	Parafrigoribacterium	NA
Actinobacteriota	Acidimicrobiia	Microtrichales	Ilumatobacteraceae	CL500-29_marine_group	NA
Actinobacteriota	Acidimicrobiia	Microtrichales	Ilumatobacteraceae	CL500-29_marine_group	NA
Actinobacteriota	Actinobacteria	Frankiales	Sporichthyaceae	hgcI_clade	NA
Actinobacteriota	Actinobacteria	Frankiales	Sporichthyaceae	NA	NA
Actinobacteriota	Actinobacteria	Micrococcales	Microbacteriaceae	Marisediminicola	antarctica
Actinobacteriota	Actinobacteria	Frankiales	Sporichthyaceae	Candidatus_Planktophila	NA
Actinobacteriota	Actinobacteria	Frankiales	Sporichthyaceae	hgcI_clade	NA
Actinobacteriota	Actinobacteria	Frankiales	Sporichthyaceae	NA	NA
Actinobacteriota	Actinobacteria	Frankiales	Sporichthyaceae	hgcI_clade	NA
Actinobacteriota	Actinobacteria	Frankiales	Sporichthyaceae	hgcI_clade	NA
Actinobacteriota	Acidimicrobiia	Microtrichales	Ilumatobacteraceae	CL500-29_marine_group	NA
Actinobacteriota	Acidimicrobiia	Microtrichales	Ilumatobacteraceae	CL500-29_marine_group	NA
Actinobacteriota	Actinobacteria	PeM15	NA	NA	NA
Actinobacteriota	Actinobacteria	Frankiales	Sporichthyaceae	hgcI_clade	NA
Actinobacteriota	Acidimicrobiia	Microtrichales	Ilumatobacteraceae	CL500-29_marine_group	NA
Actinobacteriota	Actinobacteria	Frankiales	Sporichthyaceae	hgcI_clade	NA
Actinobacteriota	Actinobacteria	Frankiales	Sporichthyaceae	Candidatus_Planktophila	NA
Actinobacteriota	Actinobacteria	Frankiales	Sporichthyaceae	hgcI_clade	NA
Actinobacteriota	Thermoleophilia	Gaiellales	NA	NA	NA
Actinobacteriota	Thermoleophilia	Solirubrobacterales	Solirubrobacteraceae	Conexibacter	NA
Actinobacteriota	Acidimicrobiia	Microtrichales	Ilumatobacteraceae	CL500-29_marine_group	NA
Actinobacteriota	Thermoleophilia	Gaiellales	NA	NA	NA
Actinobacteriota	Actinobacteria	PeM15	NA	NA	NA
Actinobacteriota	Actinobacteria	Frankiales	Sporichthyaceae	hgcI_clade	NA

Actinobacteriota	Thermoleophilia	Solirubrobacterales	Solirubrobacteraceae	NA	NA
Actinobacteriota	Acidimicrobiia	Microtrichales	Ilumatobacteraceae	CL500-29_marine_group	NA
Actinobacteriota	Actinobacteria	Frankiales	Sporichthyaceae	Candidatus_Planktophila	NA
Actinobacteriota	Acidimicrobiia	IMCC26256	NA	NA	NA
Actinobacteriota	Acidimicrobiia	Microtrichales	NA	NA	NA
Actinobacteriota	Actinobacteria	Frankiales	Sporichthyaceae	hgcI_clade	NA
Actinobacteriota	Actinobacteria	Frankiales	Sporichthyaceae	hgcI_clade	NA
Actinobacteriota	Actinobacteria	Frankiales	Sporichthyaceae	hgcI_clade	NA
Acidobacteriota	Vicinamibacteria	Vicinamibacterales	Vicinamibacteraceae	NA	NA
Acidobacteriota	Acidobacteriae	Paludibaculum	NA	NA	NA
Acidobacteriota	Acidobacteriae	Bryobacterales	Bryobacteraceae	Bryobacter	NA
Acidobacteriota	Acidobacteriae	Paludibaculum	NA	NA	NA

Supplementary Table S6. Features (or biomarkers) significantly associated ice or water. Taxonomy is provided for each feature. When no further taxonomic information is provided, the last reported rank is the feature significantly associated with the habitat. LDA size effect, *P* value (adjusted) and significance (<0.05 = *; <0.01 = **) are reported. **a.** Biomarkers of the cryosphere. **b.** Biomarkers in free-flowing water.

**Feature (taxonomic rank)**							**LDA score**	***P***	**Significance**
**Kingdom**	**Phylum**	**Class**	**Order**	**Family**	**Genus**	**Species**			
a. Enriched in the cryosphere									
Bacteria	Bacteroidota	Bacteroidia					5.11	0.002984914	**
Bacteria	Proteobacteria	Gammaproteobacteria	Burkholderiales	Comamonadaceae	Polaromonas	Polaromonas	5.10	0.004508698	**
Bacteria	Cyanobacteria	Cyanobacteriia	Leptolyngbyales	Leptolyngbyaceae	Leptolyngbyaceae		5.06	0.024018673	*
Bacteria	Cyanobacteria	Cyanobacteriia	Leptolyngbyales	Leptolyngbyaceae	Leptolyngbyaceae	Leptolyngbyaceae	5.06	0.024018673	*
Bacteria	Proteobacteria	Gammaproteobacteria	Burkholderiales	Comamonadaceae	Polaromonas		5.05	0.006706276	**
Bacteria	Bacteroidota	Bacteroidia	Cytophagales				5.02	0.001945774	**
Bacteria	Bacteroidota						5.00	0.004508698	**
Bacteria	Bacteroidota	Bacteroidia	Cytophagales	Hymenobacteraceae			4.96	0.001945774	**
Bacteria	Bacteroidota	Bacteroidia	Cytophagales	Hymenobacteraceae	Hymenobacter		4.96	0.001945774	**
Bacteria	Actinobacteriota	Actinobacteria	Micrococcales	Microbacteriaceae			4.90	0.001945774	**
Bacteria	Actinobacteriota	Actinobacteria	Micrococcales				4.89	0.001945774	**
Bacteria	Deinococcota						4.87	0.001923497	**
Bacteria	Deinococcota	Deinococci					4.87	0.001923497	**
Bacteria	Deinococcota	Deinococci	Deinococcales				4.87	0.001923497	**
Bacteria	Deinococcota	Deinococci	Deinococcales	Deinococcaceae			4.87	0.001923497	**
Bacteria	Deinococcota	Deinococci	Deinococcales	Deinococcaceae	Deinococcus		4.86	0.001923497	**
Bacteria	Deinococcota	Deinococci	Deinococcales	Deinococcaceae	Deinococcus	Deinococcus	4.86	0.001923497	**
Bacteria	Actinobacteriota	Actinobacteria	Micrococcales	Microbacteriaceae	Parafrigoribacterium		4.85	0.001945774	**
Bacteria	Actinobacteriota	Actinobacteria	Micrococcales	Microbacteriaceae	Parafrigoribacterium	Parafrigoribacterium	4.85	0.001945774	**
Bacteria	Proteobacteria	Alphaproteobacteria					4.69	0.038867104	*
Bacteria	Cyanobacteria	Cyanobacteriia	Pseudanabaenales				4.69	0.045330814	*
Bacteria	Cyanobacteria	Cyanobacteriia	Pseudanabaenales	Pseudanabaenaceae			4.69	0.045330814	*
Bacteria	Cyanobacteria	Cyanobacteriia	Pseudanabaenales	Pseudanabaenaceae	PseudanabaenaPCC-7429		4.68	0.045330814	*
Bacteria	Cyanobacteria	Cyanobacteriia	Pseudanabaenales	Pseudanabaenaceae	PseudanabaenaPCC-7429	frigida	4.66	0.045330814	*
Bacteria	Bacteroidota	Bacteroidia	Cytophagales	Hymenobacteraceae	Hymenobacter	frigidus	4.66	0.001945774	**
Bacteria	Bacteroidota	Bacteroidia	Cytophagales	Hymenobacteraceae	Hymenobacter	Hymenobacter	4.65	0.001945774	**
Bacteria	Proteobacteria	Gammaproteobacteria	Burkholderiales	Oxalobacteraceae			4.63	0.009823275	**
Bacteria	Proteobacteria	Alphaproteobacteria	Sphingomonadales				4.51	0.020136752	*
Bacteria	Proteobacteria	Alphaproteobacteria	Sphingomonadales	Sphingomonadaceae			4.51	0.020136752	*
Bacteria	Bacteroidota	Bacteroidia	Sphingobacteriales	Sphingobacteriaceae	Pedobacter	Pedobacter	4.50	0.001945774	**
Bacteria	Bacteroidota	Bacteroidia	Sphingobacteriales	Sphingobacteriaceae	Pedobacter		4.50	0.001945774	**
Bacteria	Bacteroidota	Bacteroidia	Sphingobacteriales	Sphingobacteriaceae			4.50	0.001945774	**
Bacteria	Bdellovibrionota						4.50	0.020136752	*
Bacteria	Bacteroidota	Bacteroidia	Sphingobacteriales				4.47	0.006706276	**
Bacteria							4.41	0.008786951	**
Bacteria	Proteobacteria	Alphaproteobacteria	Sphingomonadales	Sphingomonadaceae	Polymorphobacter		4.36	0.045151108	*
Bacteria	Proteobacteria	Alphaproteobacteria	Sphingomonadales	Sphingomonadaceae	Polymorphobacter	Polymorphobacter	4.36	0.045151108	*
Bacteria	Bdellovibrionota	Oligoflexia					4.35	0.028185802	*
Bacteria	Proteobacteria	Alphaproteobacteria	Acetobacterales				4.28	0.020136752	*
Bacteria	Proteobacteria	Alphaproteobacteria	Acetobacterales	Acetobacteraceae			4.28	0.020136752	*
Bacteria	Proteobacteria	Alphaproteobacteria	Acetobacterales	Acetobacteraceae	Acidiphilium		4.28	0.026614832	*

Bacteria	Proteobacteria	Alphaproteobacteria	Acetobacterales	Acetobacteraceae	Acidiphilium	Acidiphilium	4.28	0.026614832	*
Bacteria	Bdellovibrionota	Oligoflexia	0319-6G20				4.27	0.009823275	**
Bacteria	Bdellovibrionota	Oligoflexia	0319-6G20	0319-6G20			4.27	0.009823275	**
Bacteria	Bdellovibrionota	Oligoflexia	0319-6G20	0319-6G20	0319-6G20f		4.27	0.009823275	**
Bacteria	Bdellovibrionota	Oligoflexia	0319-6G20	0319-6G20	0319-6G20f	0319-6G20f	4.27	0.009823275	**
Bacteria	Bacteroidota	Bacteroidia	Cytophagales	Spirosomaceae	Arcicella	Arcicella	4.18	0.003114943	**
Bacteria	Bacteroidota	Bacteroidia	Cytophagales	Spirosomaceae	Arcicella		4.18	0.004079827	**
Bacteria	Cyanobacteria	Cyanobacteriia	Leptolyngbyales	Leptolyngbyaceae	PhormidesmisANT.L52.6	PhormidesmisANT.L52.6	4.04	0.035171785	*
Bacteria	Cyanobacteria	Cyanobacteriia	Leptolyngbyales	Leptolyngbyaceae	PhormidesmisANT.L52.6		4.04	0.035171785	*
b. Enriched in water									
Bacteria	Planctomycetota						5.17	0.004508698	**
Bacteria	Actinobacteriota	Actinobacteria	Frankiales	Sporichthyaceae			5.13	0.001857638	**
Bacteria	Actinobacteriota	Actinobacteria	Frankiales				5.13	0.001945774	**
Bacteria	Chloroflexi	Anaerolineae					5.13	0.004464706	**
Bacteria	Chloroflexi	Anaerolineae	Anaerolineales	Anaerolineaceae	Anaerolineaceae	Anaerolineaceae	5.13	0.004334202	**
Bacteria	Chloroflexi	Anaerolineae	Anaerolineales	Anaerolineaceae			5.13	0.004334202	**
Bacteria	Chloroflexi	Anaerolineae	Anaerolineales				5.13	0.004334202	**
Bacteria	Chloroflexi	Anaerolineae	Anaerolineales	Anaerolineaceae	Anaerolineaceae		5.12	0.004334202	**
Bacteria	Chloroflexi						5.08	0.009823275	**
Bacteria	Planctomycetota	Phycisphaerae					4.88	0.006706276	**
Bacteria	Planctomycetota	Phycisphaerae	Phycisphaerales	Phycisphaeraceae	CL500-3		4.88	0.006706276	**
Bacteria	Planctomycetota	Phycisphaerae	Phycisphaerales	Phycisphaeraceae	CL500-3	CL500-3	4.88	0.006706276	**
Bacteria	Planctomycetota	Phycisphaerae	Phycisphaerales				4.88	0.006706276	**
Bacteria	Planctomycetota	Phycisphaerae	Phycisphaerales	Phycisphaeraceae			4.88	0.006706276	**
Bacteria	Actinobacteriota	Actinobacteria	Frankiales	Sporichthyaceae	CandidatusPlanktophila	CandidatusPlanktophila	4.86	0.004334202	**
Bacteria	Actinobacteriota	Actinobacteria	Frankiales	Sporichthyaceae	CandidatusPlanktophila		4.86	0.004334202	**
Bacteria	Planctomycetota	Planctomycetes					4.86	0.004508698	**
Bacteria	Actinobacteriota	Acidimicrobiia					4.74	0.006646162	**
Bacteria	Actinobacteriota	Acidimicrobiia	Microtrichales				4.72	0.006646162	**
Bacteria	Cyanobacteria	Cyanobacteriia	Synechococcales	Cyanobiaceae	CyanobiumPCC-6307		4.72	0.01573409	*
Bacteria	Cyanobacteria	Cyanobacteriia	Synechococcales	Cyanobiaceae			4.72	0.01573409	*
Bacteria	Cyanobacteria	Cyanobacteriia	Synechococcales	Cyanobiaceae	CyanobiumPCC-6307	CyanobiumPCC-6307	4.72	0.01573409	*
Bacteria	Cyanobacteria	Cyanobacteriia	Synechococcales				4.72	0.01573409	*
Bacteria	Actinobacteriota	Acidimicrobiia	Microtrichales	Ilumatobacteraceae			4.71	0.006646162	**
Bacteria	Actinobacteriota	Acidimicrobiia	Microtrichales	Ilumatobacteraceae	CL500-29marinegroup		4.71	0.006646162	**
Bacteria	Actinobacteriota	Acidimicrobiia	Microtrichales	Ilumatobacteraceae	CL500-29marinegroup	CL500-29marinegroup	4.71	0.006646162	**
Bacteria	Actinobacteriota	Actinobacteria	Frankiales	Sporichthyaceae	hgcIclade	hgcIclade	4.68	0.001857638	**
Bacteria	Actinobacteriota	Actinobacteria	Frankiales	Sporichthyaceae	hgcIclade		4.68	0.001857638	**
Bacteria	Planctomycetota	Planctomycetes	Gemmatales				4.67	0.020136752	*
Bacteria	Planctomycetota	Planctomycetes	Gemmatales	Gemmataceae			4.67	0.020136752	*
Bacteria	Planctomycetota	Planctomycetes	Gemmatales	Gemmataceae	Gemmataceae		4.59	0.002953317	**
Bacteria	Planctomycetota	Planctomycetes	Gemmatales	Gemmataceae	Gemmataceae	Gemmataceae	4.59	0.002953317	**
Bacteria	Bacteroidota	Kapabacteria	Kapabacteriales				4.39	0.001730256	**
Bacteria	Bacteroidota	Kapabacteria	Kapabacteriales	Kapabacteriales	Kapabacterialesf	Kapabacterialesf	4.39	0.001730256	**
Bacteria	Bacteroidota	Kapabacteria	Kapabacteriales	Kapabacteriales	Kapabacterialesf		4.39	0.001730256	**
Bacteria	Bacteroidota	Kapabacteria					4.39	0.001730256	**
Bacteria	Bacteroidota	Kapabacteria	Kapabacteriales	Kapabacteriales			4.39	0.001730256	**
Bacteria	Planctomycetota	Planctomycetes	Pirellulales	Pirellulaceae			4.35	0.006467554	**
Bacteria	Planctomycetota	Planctomycetes	Pirellulales				4.35	0.006467554	**
Bacteria	Acidobacteriota						4.27	0.004508698	**

Bacteria	Verrucomicrobiota						4.23	0.038867104	*
Bacteria	Verrucomicrobiota	Verrucomicrobiae					4.22	0.028185802	*
Bacteria	Planctomycetota	Planctomycetes	Pirellulales	Pirellulaceae	Pirellulaceae	Pirellulaceae	4.21	0.006118156	**
Bacteria	Planctomycetota	Planctomycetes	Pirellulales	Pirellulaceae	Pirellulaceae		4.21	0.006118156	**
Bacteria	Actinobacteriota	Actinobacteria	Frankiales	Sporichthyaceae	Sporichthyaceae		4.20	0.013127303	*
Bacteria	Actinobacteriota	Actinobacteria	Frankiales	Sporichthyaceae	Sporichthyaceae	Sporichthyaceae	4.20	0.013127303	*
Bacteria	Gemmatimonadota						4.20	0.001945774	**
Bacteria	Gemmatimonadota	Gemmatimonadetes					4.20	0.001945774	**
Bacteria	Gemmatimonadota	Gemmatimonadetes	Gemmatimonadales				4.20	0.001945774	**
Bacteria	Gemmatimonadota	Gemmatimonadetes	Gemmatimonadales	Gemmatimonadaceae			4.20	0.001945774	**
Bacteria	Acidobacteriota	Vicinamibacteria					4.08	0.001730256	**
Bacteria	Proteobacteria	Gammaproteobacteria	Burkholderiales	Comamonadaceae	Limnohabitans	Limnohabitans	4.08	0.001730256	**
Bacteria	Proteobacteria	Gammaproteobacteria	Burkholderiales	Comamonadaceae	Limnohabitans		4.08	0.001730256	**
Bacteria	Proteobacteria	Gammaproteobacteria	Burkholderiales	Comamonadaceae	Polaromonas	Polaromonaseurypsychrophila	4.08	0.001857638	**
Bacteria	Acidobacteriota	Vicinamibacteria	Vicinamibacterales				4.07	0.001730256	**
Bacteria	Gemmatimonadota	Gemmatimonadetes	Gemmatimonadales	Gemmatimonadaceae	Gemmatimonas		4.02	0.001945774	**
Bacteria	Gemmatimonadota	Gemmatimonadetes	Gemmatimonadales	Gemmatimonadaceae	Gemmatimonas	Gemmatimonas	4.02	0.001945774	**
Bacteria	Acidobacteriota	Vicinamibacteria	Vicinamibacterales	Vicinamibacteraceae	Vicinamibacteraceae	Vicinamibacteraceae	4.02	0.002391202	**
Bacteria	Acidobacteriota	Vicinamibacteria	Vicinamibacterales	Vicinamibacteraceae			4.02	0.002391202	**
Bacteria	Acidobacteriota	Vicinamibacteria	Vicinamibacterales	Vicinamibacteraceae	Vicinamibacteraceae		4.02	0.002391202	**

Supplementary Table S7. Source tracking of bacterial taxa in the glacier-lake-outflow continuum. Sinks are the lake (including the glacier-lake interface and sampling depths), lake ice (surface ice in contact with air, and bottom ice in contact with water column) and outflow (mouth of Thores River at the lake, and junction with Disraeli Fiord, 16 km downstream). Potential sources are the glacier, lake ice (for the Thores Lake and river sinks) and lake water (for the lake ice and river sinks). Taxa that could not be sourced to these categories are marked as "unknown". Proportions are presented as averages over rarefactions (standard deviation in parentheses) (10 Gibbs sampling draws), dashes indicate not applicable.

Sinks		Sources (% origin)			
		Unknown	Glacier	Lake ice	Lake
Lake	Interface	53.4 (±1.6)	0.90 (±0.27)	46.7 (±1.5)	-
	5m	13.2 (±0.6)	0.09 (±0.07)	86.8 (±0.5)	-
	5m	7.3 (±0.2)	0.11 (±0.09)	92.6 (±0.1)	-
	15m	11.0 (±0.8)	0.16 (±0.10)	88.8 (±0.8)	-
	17m	7.7 (±0.3)	0.22 (±0.10)	92.1 (±0.3)	-
	40m	8.6 (±0.2)	0.21 (±0.10)	91.2 (±0.3)	-
Lake ice	Surface (contact with air)	7.5 (±0.4)	91.9 (±0.2)	-	0.56 (±0.22)
	Bottom (contact with water)	4.3 (±0.2)	33.1 (±0.8)	-	62.6 (±0.72)
Outflow	Mouth of Thores River	10.1 (±0.2)	0.15 (±0.05)	2.0 (±0.7)	87.7 (±0.7)
	River/Disraeli Fiord interface	52.3 (±2.2)	1.52 (±0.67)	14.8 (±1.7)	31.5 (±2.3)

SUPPLEMENTARY FIGURES

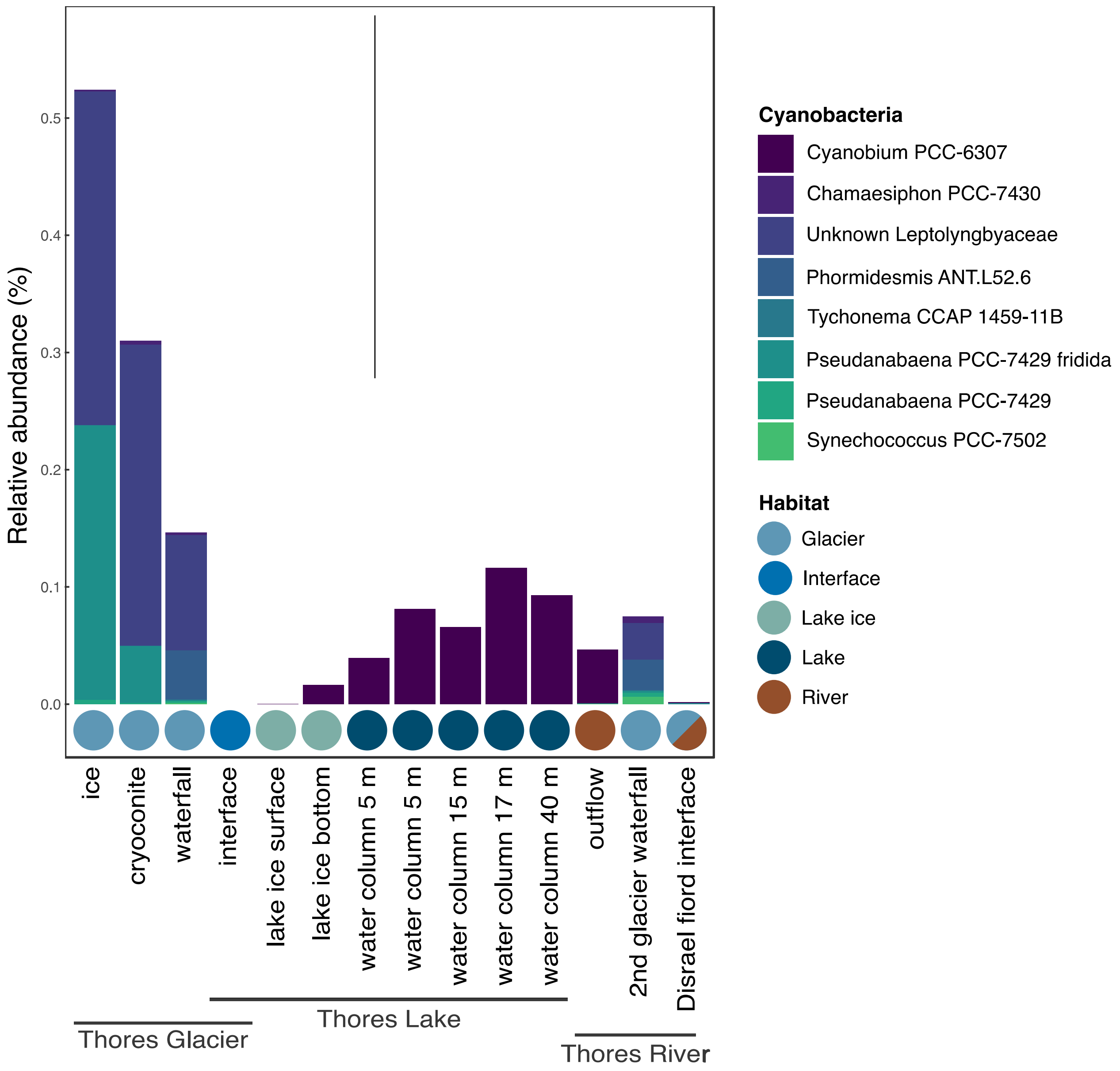

Supplementary Fig. S1 Cyanobacteria in the Thores glacier-lake-outflow continuum. Relative abundance of the main representatives of the Cyanobacteria phylum.

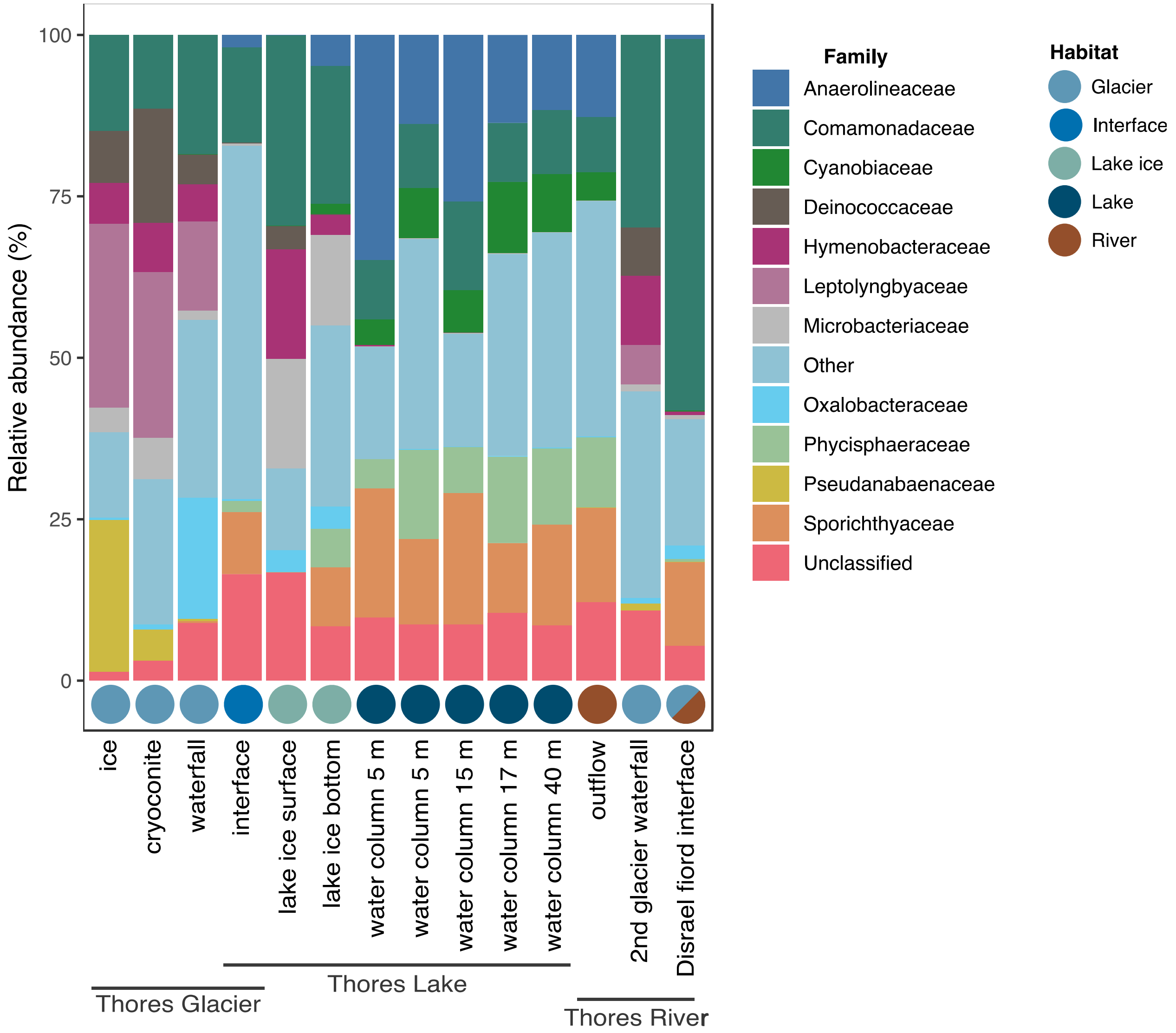

Supplementary Fig. S2 Family composition in the Thores glacier-lake-outflow continuum. Family-level relative abundance (top dataset-wide families shown, the rest grouped in “Others”). Colors show habitat type – when a sample was taken at the interface of two categories, both colors are represented.

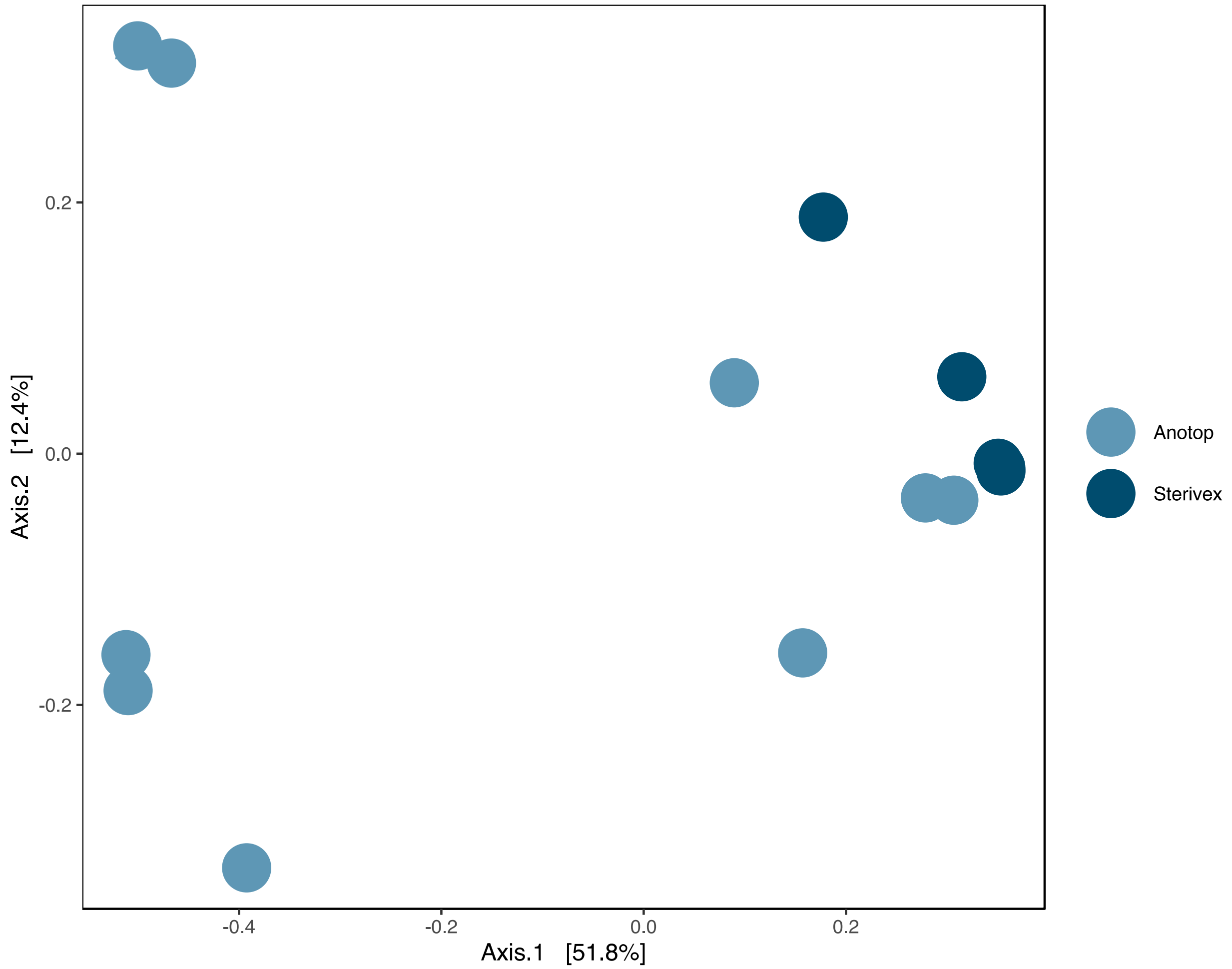

Supplementary Fig. S3 Effect of filter type on Bray-Curtis dissimilarity. While filter type had a significant effect on community composition (P = 0.01, R^2 = 25%), lake water filtered onto Sterivex most resembled Anotop-filtered water samples. The effect of habitat was also greater than that of filters (Figure 3d).
